# Pleomorphism and drug resistant cancer stem cells are characteristic of aggressive primary meningioma cell lines

**DOI:** 10.1186/s12935-017-0441-7

**Published:** 2017-07-21

**Authors:** Ishaq Khan, Saleh Baeesa, Mohammed Bangash, Hans-Juergen Schulten, Fahad Alghamdi, Hanadi Qashqari, Nawal Madkhali, Angel Carracedo, Mohamad Saka, Awatif Jamal, Jaudah Al-Maghrabi, Mohammed AlQahtani, Saleh Al-Karim, Ghazi Damanhouri, Kulvinder Saini, Adeel Chaudhary, Adel Abuzenadah, Deema Hussein

**Affiliations:** 1 King Fahd Medical Research Center, King Abdulaziz University, P.O. Box. 80216, Jeddah, 21589 Saudi Arabia; 2 Department of Biological Sciences, Faculty of Science, King Abdulaziz University, Jeddah, 21589 Saudi Arabia; 3 Center of Excellence in Genomic Medicine Research, King Abdulaziz University, Jeddah, 21589 Saudi Arabia; 4 Centre of Innovation for Personalized Medicine, King Abdulaziz University, Jeddah, 21589 Saudi Arabia; 5 Division of Neurosurgery, King Abdulaziz University, Jeddah, 21589 Saudi Arabia; 6 Pathology Department, King Abdulaziz University, Jeddah, 21589 Saudi Arabia; 7 Galician Foundation of Genomic Medicine, Cyber-University of Santiago de Compostela, 15706 Santiago De Compostela, Spain; 8 School of Biotechnology, Eternal University, Baru Sahib Road, Sirmour, 173101 Himachal Pradesh India

**Keywords:** Meningioma, Cancer stem cells, Caspase 3, Nestin, Ki67, CD133, Sox2, AGR2, Frizzled 9, Vimentin, Cisplatin, Etoposide, Drug resistance

## Abstract

**Background:**

Meningioma tumors arise in arachnoid membranes, and are the most reported central nervous system (CNS) tumors worldwide. Up to 20% of grade I meningioma tumors reoccur and currently predictive cancer stem cells (CSCs) markers for aggressive and drug resistant meningiomas are scarce.

**Methods:**

Meningioma tissues and primary cell lines were investigated using whole transcriptome microarray analysis, immunofluorescence staining of CSCs markers (including CD133, Sox2, Nestin, and Frizzled 9), and drug treatment with cisplatin or etoposide.

**Results:**

Unsupervised hierarchical clustering of six meningioma samples separated tissues into two groups. Analysis identified stem cells related pathways to be differential between the two groups and indicated the de-regulation of the stem cell associated genes *Reelin (RELN), Calbindin 1 (CALB1) and Anterior Gradient 2 Homolog (AGR2)*. Immunofluorescence staining for four tissues confirmed stemness variation in situ. Biological characterization of fifteen meningioma primary cell lines concordantly separated cells into two functionally distinct sub-groups. Pleomorphic cell lines (NG type) grew significantly faster than monomorphic cell lines (G type), had a higher number of cells that express Ki67, and were able to migrate aggressively in vitro. In addition, NG type cell lines had a lower expression of nuclear Caspase-3, and had a significantly higher number of CSCs co-positive for CD133+ Sox2+ or AGR2+ BMI1+. Importantly, these cells were more tolerant to cisplatin and etoposide treatment, showed a lower level of nuclear Caspase-3 in treated cells and harbored drug resistant CSCs.

**Conclusion:**

Collectively, analyses of tissues and primary cell lines revealed stem cell associated genes as potential targets for aggressive and drug resistant meningiomas.

**Electronic supplementary material:**

The online version of this article (doi:10.1186/s12935-017-0441-7) contains supplementary material, which is available to authorized users.

## Background

Meningiomas are leptomeningeal neoplasms thought to originate from arachnoid membranes that form the cranial and spinal meninges [1]. They account for the most World Health Organization (WHO) classified Central Nervous System (CNS) tumors in the USA [2], and are highly reported worldwide [3]. WHO classifies meningiomas into fifteen variants with grade I–III. Although grade III variants are most at risk for recurrence, a portion of grade I tumors reoccur (up to 20%) and currently there are very few predictive makers for such group [4–6]. One of the first chromosomal mutation identified for meningiomas was the deletion of the long arm of chromosome 22 [7]. In particular, a role of the *type 2 neurofibromatosis (NF2)/Merlin* gene located on 22q12 was implicated [8]. Genomic analysis of meningiomas tissues identified deregulations in the oncogenic genes *Phosphoinositide 3*-*kinase (PIK), RAC*-*alpha serine/threonine*-*protein kinase 1 (AKT1), the G protein*-*coupled receptor smoothened (SMO), TNF receptor*-*associated factor 7 (TRAF7), and Kruppel*-*like factor (KLF4)* [9–11].

Deregulated molecular pathways identified based on bulk tumor analyses, have been utilized to predict prognosis and help determine appropriate targeted therapy [12]. However, despite all recent development in targeted therapy, surgery and radiation therapy remain typically the main methods of treatment for meningioma, even though both pose post-treatment challenges depending upon tumor location [13]. For several CNS tumors, clinical trials that test for combinations of standard chemotherapeutic agents are still under progress. Cisplatin and etoposide are standard chemotherapeutic agents for many tumors. Both drugs alone, in combination with each other, or in combination with other drugs were found to be effective in adult and pediatric patients with low and high grade gliomas [14–17]. These drugs are not primarily cell cycle affected, thus they could potentially be useful for the slow growing meningioma cells [18]. Preclinical determination of the degree of resistance of meningiomas to either drugs and the identification of associated markers may prove valuable in improving tumor grading and prognosis [19].

A crucial component for the development of targeted therapy is the availability of live tumor models [20]. Early passaged cell cultures represent the “Central dogma” of patients’ tumors more faithfully than long term commercial cultures, yet very few attempts have been made to use such cells for pharmacological testing in meningiomas [21]. Growth associated receptors and cancer stem cells (CSCs), cancer cells that express stem cell markers and are highly tolerant to adverse growth conditions, have been identified in stable meningioma cell lines [22–31], even in cultures that have been sustained in a wide range of media conditions [27, 32, 33]. However, identified meningioma CSCs in primary cell lines are not commonly associated with drug resistance, possibly because such studies require abundant number of cells which is often restricted by the size of excised tissues [34].

Previously we published the gene expression profiles for meningioma patients tissues collected for our cohort [35]. For this work, we focused on examining the properties of the corresponding meningioma primary cell lines in relation to biology, CSCs and drug resistance.

## Methods

### RNA extraction, microarray processing and gene expression analysis

Tumor tissue RNA were isolated and processed for microarray and gene expression analysis as previously described [35], and data was deposited at the NCBI’s Gene Expression Omnibus under accession number GSE77259. For gene expression analysis, based on differentially expressed probe sets, an unsupervised clustering was performed for Jed36_MN, Jed49_MN Jed04_MN, Jed18_MN, Jed34_MN, and Jed40_MN and visualized in a hierarchical clustering graph containing a colored heat map and adjunct dendrograms for displaying expression values and distance metrics between objects (samples or probe sets), respectively. Biological significance of expression data was interpreted by employing the core functional analysis workflow of the Ingenuity Pathways Analysis Software (IPA) (Ingenuity Systems, Redwood City, CA, USA). The Ingenuity Knowledge Base served as reference data set. Significance of relationships between data set molecules and functional frameworks, e.g. canonical pathways, provided by IPA was indicated by Fisher’s exact test p values.

### Meningioma cell cultures initiation

Meningioma specimens collected between February 2013 and December 2015 were obtained within 30 min of tumor removal and distributed into both RNAlater solution (Life Technologies) for RNA/DNA extraction and cell culture using mechanical and enzymatic dissociation methods [36]. For cell culture initiation, surgical specimens were minced, dissociated with a scalpel in Hank’s Balanced Salt Solution (HBSS) and further incubated with 1× enzyme solution in HBSS [0.2 mg/mL DNase type 1 (2000 U/mg), Sigma; 0.4 mg/mL collagenase type 1a (125 U/mg), Gibco; HBSS 10 mL (calcium and magnesium free), Invitrogen]. Samples were rotated for 15 min and then were centrifuged at 1000 rpm (180×*g*) for 3 min at room temperature, re-suspended in DMEM-F12 (Gibco), 10% FBS (HyClone), 100 U/mL penicillin, and 100 µg/mL streptomycin, and maintained in standard humidified incubators at 5% CO_2_. After 24 h, non-adherent cells were removed by washing with Phosphate Buffered Saline (PBS; HyClone) and the adherent cells were cultured until they reached confluence, then split (1:2) every 12–30 days dependent on the cell line. For each of tumors Jed62_MN and Jed79_MN, tissues were divided into four representative portions and each was placed into a 25 cm^2^ tissue culture flasks containing a type of media (DMEM-F12 and 10% FBS, or DMEM high glucose concentrations of 4500 mg/L (Gibco) with 10% FBS, or DMEM high glucose concentrations of 4500 mg/L (Gibco) with 5% FBS, or DMEM low glucose concentrations of 1000 mg/L (Gibco) with 10% FBS).

### Determination of cell morphology and trends

Cell cultures were observed for a period of 4 weeks with an inverted microscope (Leica DMI6000) to determine their morphologies. Twenty images of cells were taken at 10× magnifications each week using a digital microscope camera (Leica DFC425). The average number of counted cells per cell line exceeded 500 per week. The weekly morphology percentages were calculated as the total number of cells displaying a particular morphology, divided by the total number of cells counted. Morphological profiles for cells were confirmed by immunofluorescence staining with mouse anti-Vimentin (1:100, ab8978, abcam). All cell lines and images were reviewed independently at least twice to confirm counts.

### Immunofluorescence staining

For cell lines, cells were seeded on chamber slides and fixed with 4% Paraformaldehyde (PFA). For in situ staining, fresh frozen tissues were cut at 4 μM sections, then fixed with 4% Paraformaldehyde (PFA). The antibodies used for standard immunostaining were rabbit anti-Caspase-3 (1:100, ab4051, abcam), mouse anti-CD133 (1:100, W6B3C1, Miltenyi), rabbit anti-Sox2 (1:200, 09-0024, Miltenyi), mouse anti Sox2 (1:100, ab75485, abcam), mouse anti Nestin (1:50, ab6142, abcam), rabbit anti-Ki67 (1:200, ab16667, abcam), mouse anti-Vimentin (1:100, ab8978, abcam), rabbit anti- Frizzled 9 (1:100, ab150515, abcam), mouse anti-BMI1 proto-oncogene, polycomb ring finger (BMI1) antibody (1:100, ab14389, abcam), and rabbit Anti-Anterior Gradient 2 antibody (1:100, ab76473, abcam). For secondary goat antibodies, 488 anti-mouse (1:300, ab150105, abcam) and 555 anti-Rabbit (1:700, ab150074, abcam) were used. Pictures were taken at 20× magnifications using Leica DMI6000 microscope and Leica DFC425 camera. Photos were edited in Photoshop 7.0 and signal levels were compared to negative controls of secondary only. *T* test or Chiχ^2^ values were retrieved using Statistical Package for the Social Sciences (SPSS) Graduate Pack 21.0.

### Growth inhibition assays

To determine IC_50_ values for cisplatin and etoposide, preliminary growth inhibition experiments were completed for three cell lines that had fast growth capability (Jed38_MN, Jed45_MN, Jed49_MN), (Additional file 1: Figure S1). Cells were plated in 96-well plates at 5000 cells per well supplemented with DMEM-F12 and 10% FBS and left to adhere overnight at 37 °C in 5% CO_2_. Following attachment, cells were treated for 2 h with cisplatin (in Saline) or etoposide (in DMSO) at increasing concentrations of drug. Twelve days following treatment, cells were fixed with 4% paraformaldehyde (PFA) and stained with crystal violet. 10% acetic acid was added to cells and absorbance was measured at 590 nm. The survival fractions (the absorbance values of the test well expressed as a percentage of the untreated control) were calculated. All assays were performed in triplicate.

## Results

### Clinical characteristic and gene expression features of tumors’ tissues

**Fig. 1:**
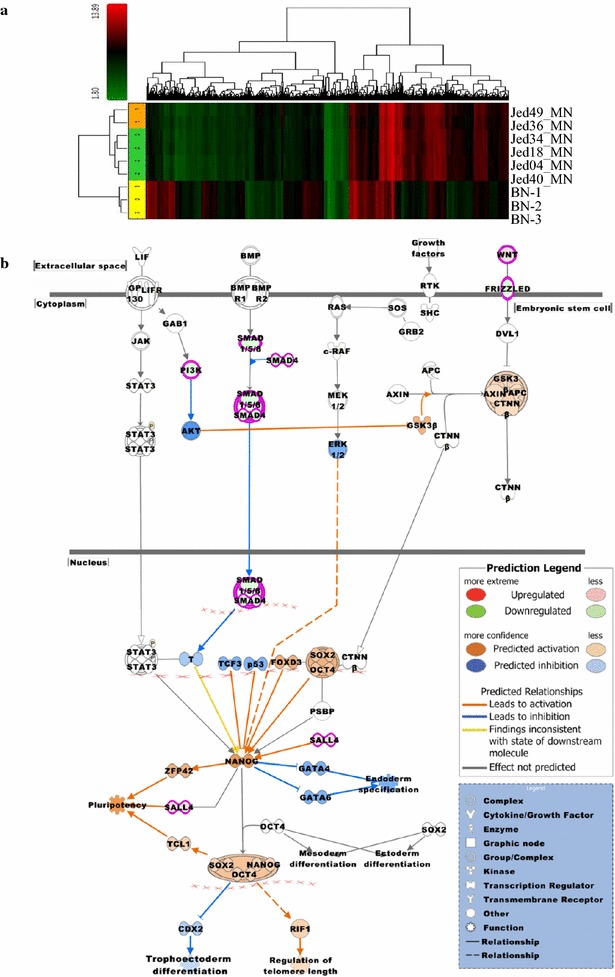
Gene expression profiles for six meningioma tissues. **a** Unsupervised hierarchical clustering showing Jed49_MN and Jed36_MN (group 1) tumors cluster separately to Jed04_MN, Jed18_MN, Jed34_MN and Jed40_MN (group 2). The latter are closer to normal brain gene expression (BN-1, BN-2 and BN-3) than Jed49_MN and Jed36_MN tumors. Cluster analysis shows 4472 differentially expressed probe sets (p < 0.005 and fold change >2.0). Color scheme for expression levels: red for comparably higher and green for comparably lower expression values. **b** The canonical pathway ‘Role of NANOG in Mammalian Embryonic Stem Cell Pluripotency’ was identified as one of the top pathways related to a list of 404 probe sets which were differentially expressed between group 1 and group 2. The pathway was overlaid with the molecule activity predictor to pre-calculate further molecular effects, as outlined in the prediction legend

Table 1 shows the clinical profiles for patients from whom the fifteen meningioma primary cell lines were derived. Additional file 2: Figure S2 shows H&E representative sections of histological variants of meningiomas included in this work. Whole transcriptome microarray analysis was conducted for six tissues collected prior culturing that provided sufficient RNA of good quality and quantity for microarray analysis, and produced a primary cell line. Unsupervised hierarchical clustering grouped tumors into two sub-groups, group 1 or group 2, Fig. 1a. Gene expression profiles showed 180 probe sets that were up- and 223 probe sets were down-regulated in group 1 verses group 2 type meningiomas (P < 0.05 and fold change >2.0), Additional file 3: Table S1. The respective numbers were 2520 up- and 2436 down-regulated probe sets for group 1 verses normal brain (BN) and 2744 up- and 2529 down-regulated probe sets for group 2 verses BN. Importantly, the main differential pathways between the two groups appeared to be stem cell related, Fig. 1b and Additional file 4: Table S2. The top identified differential pathways that were up-regulated in group 1 tumors included MSP-RON Signaling Pathway (P 3.38E−03), the Role of NANOG in Mammalian Embryonic Stem Cell Pluripotency Pathway (P 3.96E−03) and Human Embryonic Stem Cell Pluripotency (P 9.73E−03). Within the NANOG pathway, *Spalt*-*Like Transcription Factor 4 (SALL4)* (fold change: 3.570) and *Wingless*-*type MMTV Integration Site Family, member 6 (WNT6)* (fold change: 2.340) were deregulated. For individual genes, the top four up-regulated molecules in group 1 tumors were Reelin (RELN) (fold change: 27.130; P 0.007), *Calbindin 1 (CALB1)* (fold change: 23.540; P 0.047), *Killer Cell Lectin*-*Like Receptor Subfamily C, Member 4 (KLRC4)* (fold change: 13.670; P 0.033), and *Anterior Gradient 2 Homolog (AGR2)* (fold change: 12.660; P 0.029). The top four down-regulated molecules in group 1 tumors were *Cytochrome P450*, *Family 4 Subfamily B Polypeptide 1* (CYP4B1) (fold change: −17.400; P 0.004), F-*Box Protein 32 (FBXO32)* (fold change: −15.980; P 0.014), *Mesenteric Estrogen*-*Dependent Adipogenesis (MEDAG)* (fold change: −10.940; P 0.010), and *Serpin Peptidase Inhibitor, Clade A (Alpha*-*1 Antiproteinase, Antitrypsin)*, *Member 3 (SERPINA3)* (fold change: −8.950; P 0.038). Expression for the stem cell related markers Nestin, CD133, Sox2, AGR2 and the known oncogenic BMI1 protein, investigated using in situ tissue immunofluorescence for four additional tumors (Jed 62_MN, Jed40_MN, Jed49_MN and Jed45_MN), support the observed gene expression trends, Additional file 5: Figure S3 and Additional file 6: Figure S4. Overall, the expression of these markers was highest in the recurrent grade III tumor Jed45_MN.

**Table 1:** Clinical profiles for patients whom the cell lines were derived from

Cell line	Age	Gender	Sub-classification	Grade	P/R	Tumour site	Treatment
Jed04_MN	57	F	Transitional	I	P	Left posterior fossa	Surgery 39706-1
Jed09_MN	33	M	Transitional	I	P	Right parietal	Surgery 39706-1
Jed12_MN	43	F	Angiomatous	I	P	Biocoronal and subfrontal suprasellar	Surgery 39706-1
Jed18_MN	64	F	Meningothelial	I	P	Left sphenoid wing	Surgery 9007-02
Jed33_MN	73	F	Fibroblastic	I	P	Posterior fossa	Surgery 397-09-2
Jed34_MN	41	M	Meningothelial	I	P	Left sphenoid wing	Surgery 9007-02
Jed36_MN	36	M	Transitional	I	P	Supratentorial	Surgery 39706-1
Jed38_MN	46	F	Transitional	I	P	Left clinoidal	Surgery 39706-1
Jed39_MN	33	F	Meningothelial	I	P	Left subfrontal	Surgery 9007-02
Jed40_MN	64	F	Fibroblastic	I	P	Cerebellopontine angle	Surgery 9007-02
Jed43_MN	55	F	Psammomatous	I	P	Extra axial spinal	Surgery 40312-00
Jed62_MN	44	F	Transitional	I	P	Right convexity	Excision via burr holes
Jed49_MN	51	F	Fibroblastic	II	R	Superior sagittal sinuses	Surgery 39703-02
Jed79_MN	19	M	Chordoid	II	P	Right convexity	Surgery 39706-1
Jed45_MN	33	M	Metastatic rhabdoid	III	3rd R	Vertebral body	48639-00 excision of vertebra (prior treatment of carboplatin)

Surgical treatments codes indicate: 39706-1 decompression and osteoplastic craniotomy and excision, 9007-02 craniotomy, other procedure and excision, 397-09-2 removal of lesion of cerebellum, 39706-1 decompression and osteoplastic craniotomy and excision, 40312-00 removal of spinal intradural lesion, 48639-00 excision of vertebra (prior treatment of carboplatin), 39703-02 biopsy of cerebral meninges. *P/R* primary/recurrent

### Biological characterization of meningioma primary cell lines also separated cell lines into two functionally distinct sub-groups

**Fig. 2:**
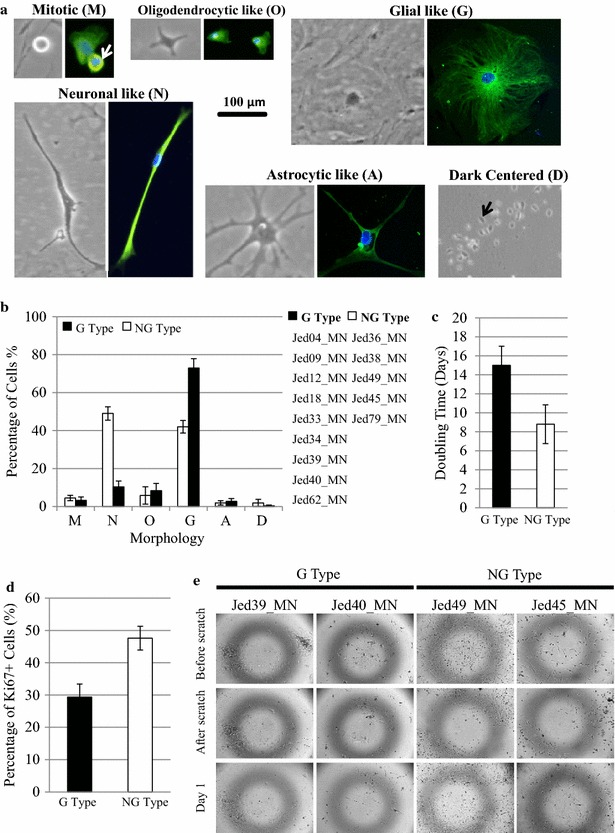
Biological characteristics of meningioma primary cell lines. **a** Observed morphological characteristics showing cells displaying different phase and Vimentin stained structures. *M Type* round, mitotic like, (10–60 µm); *N Type* neuronal-like, bipolar, spindle like, (100–500 µm in length); *O Type* flat and short spikes or processes, oligodendrocytic-like (10–100 µm); *G Type* large, cytoplasmic region, with no/few processes, glial-like (100–500 µm); *A Type* many long spikes or processes with visible central cytoplasm, astrocytic-like (100–700 µm Length of processes); and *D Type* oval and dark phase centered (20–60 µm). Images were taken at ×20 magnifications. **b** Average percentages of morphologies counts for all primary cell lines. *Horizontal accesses* show the different morphologies mentioned in **a**. Two groups emerged, cell lines that had predominantly G type cells (9 cell lines) or those that had a mix composition of N and G type cells (5 cell lines). **c** Average doubling time for G type and NG type cell lines at early passages. **d** Average percentage of Ki67 positive cells in four G and two NG cell lines. **e** An in vitro scratch assay for two G type and NG type cell lines. Cells on the edge of the newly created gap moved toward the opening to close the “scratch” within a day in NG type cell lines. Images were taken at ×5 magnifications

Consistent with differences observed in the analyzed meningioma tissues, newly initiated primary cell lines appeared to separate into two main groups with differences detect for morphological profiles, proliferation, and invasion in vitro. Following cell lines initiations, distinct cell shapes were observed in all cells lines, Fig. 2a. Cells were either round mitotic like (M type), bipolar spindle neuronal like (N type), small-flat with short spikes or processes oligodendrocytes like (O type), had large cytoplasmic regions with no or few processes and glial like (G type), were cells that had long processes with small cytoplasmic regions and astrocytes like (A type), or those that looked dead and had oval shapes and with dark inclusions (D type). Counts of the frequency of these morphologies within a cell line were initially observed to be changeable; however, frequencies appeared to stabilize after 3 weeks of culturing. To investigate whether the stabilized morphological counts were not driven solely by media conditioning, two tumors (Jed62_MN and Jed79_MN) were each divided upon arrival into four tumor representative portions, and each section was grown separately into a type of media, Additional file 7: Figure S5. Percentages of counts showed that cell morphology trends for sub-lines grown in different conditions had a consistent prominent type by week four regardless of used media type. Morphological trends were thus determined for all cell lines, and two main trends were observed. Cell lines had either prominently G type cells or a mix of N and G type cells, Fig. 2b. The exception was Jed43_MN that showed a mixed morphology trend. Interestingly, four out of five NG type cell lines came from tumor tissues observed to have a relatively high mitotic index count [Jed38_MN (11 cells/field 20×), Jed45_MN (165 cells/field 20×), Jed49_MN (33 cells/field 20×), Jed79_MN (10 cells/field 20×)]. Importantly, NG type cell lines appear to grow significantly faster than G type cell lines (T test, P 0.027) (Fig. 2c), have a higher number of cells that express Ki67 (T test, P 0.004) (Fig. 2d), and appear to be able to migrate more aggressively in vitro (Fig. 2e). In addition, clonogenic experiments indicated that while G types cells failed to form colonies within a period of 30 days, an average of 30 ± 5 clones were able to grow when plating 2000 of early passaged NG type cells.

### Cancer stem cells markers are differentially expressed in meningioma cell lines’ subgroups

**Fig. 3:**
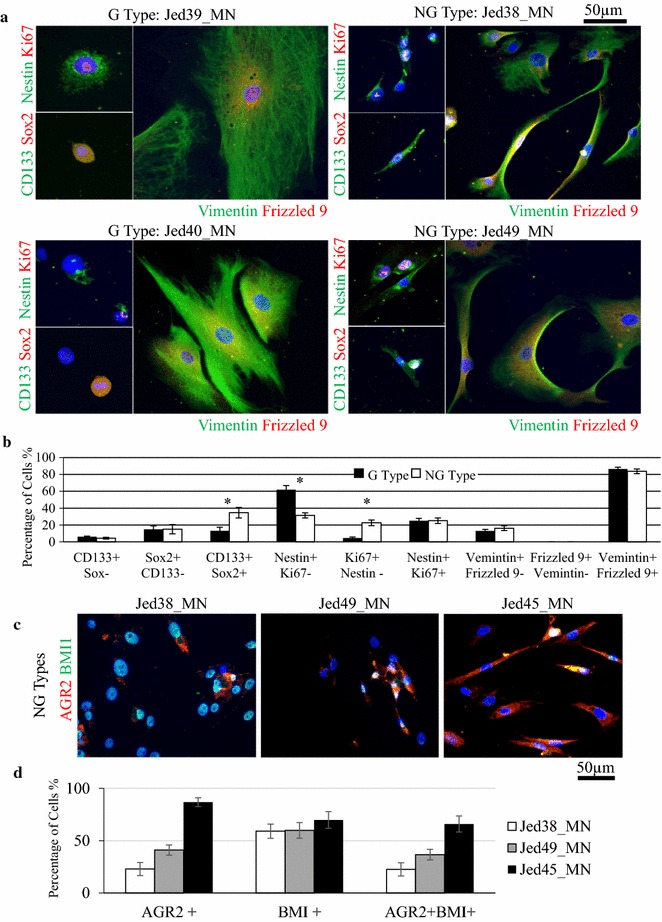
The expression of cancer stem cells markers in meningioma primary cell lines. **a** Immunofluorescence images for two G Type cell lines (Jed39_MN and Jed40_MN) and two NG Type cell lines (Jed38_MN and Jed49_MN). Images show cells co-stained positively for CD133+ Sox2+ (*green*, *red*), Nestin+ Ki67+ (*green*, *red*), or Vimentin+ Frizzled 9+ (*green*, *red*). **b** Average percentages of cells co-stained with combinational markers in G and NG cell lines. **c** High expression of AGR2 in NG cell lines. Immunofluorescence images of Jed38_MN, Jed45_MN and Jed49_MN showing co staining of AGR2 (*red*) with BMI1 (*green*). **d** Percentages of cells that express AGR2 only, or BMI1, or co-express both markers in each cell line. *Error bars* represent errors between independent counts of cell lines within a group and three independent counts were completed per cell line. *Asterisk* indicate χ^2^ significant difference

Since stem cell pathways appeared to be differential in tissues, primary G type and NG type cell lines were also investigated for differences in stemness. Immunofluorescence was used to co-stain cells for stem cell markers CD133 Sox2, Nestin Ki67, or Vimentin Frizzled 9 in four cell lines (G types: Jed39_MN and Jed40_MN, NG types: Jed38_MN and Jed49_MN). Interestingly, CSCs stained co-positive for all combinations were present in all cell lines, Fig. 3a. However, co-positivity for either CD133+ Sox2+, or for Ki67+ Nestin- was significantly higher in NG type cell lines (CD133+ Sox2+, χ^2^: 9.681, P < 0.01; Ki67+ Nestin-, χ^2^: 9.953, P < 0.01), Fig. 3b. Interestingly, the percentages of cells positive for Nestin but negative for Ki67 were significantly higher in G type cell lines (χ^2^: 12.156, P < 0.001). The expression of the recently associated stem cell protein AGR2, which was detected earlier to be deferentially expressed in our tissues, was also analyzed in NG type cell lines. Three NG type cell lines were co-stained to detect AGR2 with the oncogenic stem cell associated protein BMI1, Fig. 3c. High levels of AGR2 expression were consistently detected in these cell lines; however, expression seems to be varied with the highest levels seen in Jed45_MN cell line, Fig. 3d. The number of cells over expressing AGR2 in two G type cell lines, Jed39_MN and Jed40_MN, was also estimated, Additional file 8: Figure S6.

### NG type cell lines have a higher tolerance to cisplatin and etoposide treatment than G type cell lines

**Fig. 4:**
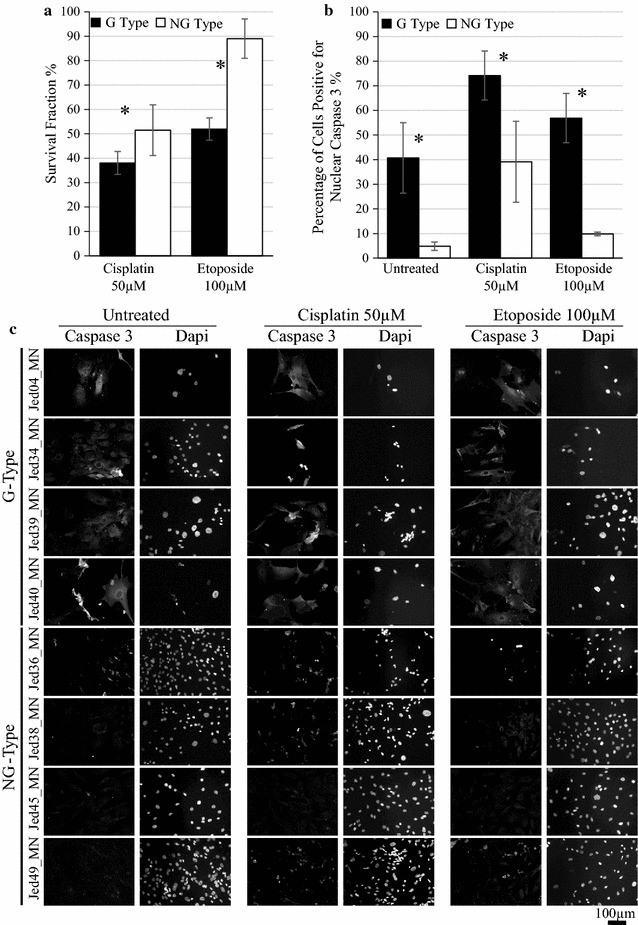
Cell lines’ tolerance to cisplatin and etoposide. **a** Survival fraction of cell lines measured 12 days following treatments. **b** The percentages of cells positive for nuclear Caspase-3 in untreated and drug treated cells 24 h following treatments. **c** Immunofluorescence images of Caspase-3 stained cells for four G Type cell lines (Jed04_MN and Jed34_MN, Jed39_MN and Jed40_MN) and four NG Type cell lines (Jed36_MN, Jed38_MN, Jed45_MN, Jed49_MN), 24 h following treatments. *Bars* indicate average counts of cell lines for each group, and error bars represent the SEM. *P < 0.05

To address whether the sub-groups had different drug response, four NG and four G type cell lines were treated with cisplatin (50 µM) or etoposide (100 µM). NG type cell lines survived treatment significantly better than G type cell lines (cisplatin, P 0.035; etoposide, P 0.009), Fig. 4a. To test whether survival was associated with resistance to initiate apoptosis, treated cells were stained with Caspase-3 a day following treatment, Fig. 4b, c. NG type cell lines had a lower expression of nuclear Caspase-3 compared to G type cell lines (untreated, P 0.024; cisplatin, P 0.012; etoposide, P 0.032).

### Cancer stem cells in NG cell lines survive cisplatin and etoposide treatment

**Fig. 5:**
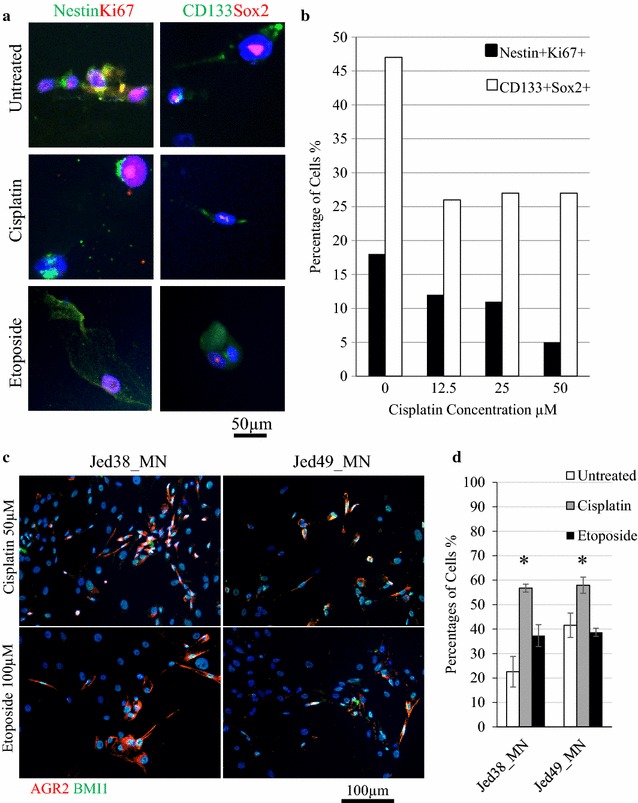
Cancer stem cells in NG cell lines survive cisplatin and etoposide treatment. **a** Immunofluorescence images of Jed49_MN survived cells positive for CD133+ Sox2+ (*green*, *red*) or Nestin+ Ki67+ (*green*, *red*). **b** Counts for survived cells treated with increasing concentrations of cisplatin and co-stained with CD133+ Sox2+ (*white bars*) or with Nestin+ Ki67+ (*black bars*). 100 cells were counted per concentration. **c** Immunofluorescence images of Jed38_MN, and Jed49_MN following treatment with either cisplatin or etoposide (100 µM) showing co-staining of AGR2 (*red*) with the oncogenic marker BMI1 (*green*). **d** Percentages of cells co-express AGR2 and BMI1 in treated cells. *Error bars* represent errors between three independent counts of 100 cells. *Asterisk* represents P < 0.05

To test whether meningioma CSCs survived treatment with cisplatin or etoposide, 12 days following treatment, treated Jed49_MN cells (NG type) were stained with CD133 and Sox2 or Nestin and Ki67. Cells co-positive for CD133+ Sox2+ or Nestin+ Ki67+ were detected following treatment with cisplatin or etoposide, Fig. 5a. For cisplatin treatment, 25% of CD133+ Sox2+ and 5% Nestin+ Ki67+ survived at 50 µM, Fig. 4b. Similarly, resistant CSCs co-expressing AGR2 and BMI1 survived treatment with cisplatin or etoposide, Fig. 5c, d.

## Discussion

We examined multiple bio-parameters for meningiomas through analysis of both eight tissue samples and 15 primary cell lines. Whole transcriptome microarray analysis for six tumor samples identified two groups of meningiomas with differential stem cell related pathways and a number of novel stem cell related biomarkers, including AGR2. Biological characterization of primary meningioma cell lines also sub-grouped cell lines into two main types; G type with predominantly glial like cells that grow slowly and were less viable, and NG type that had a mix of neuronal like and glial like cells, grow faster and showed invasive properties. In addition, NG type cells had a significantly higher percentage of cancer stem cells that express CD133+ Sox2+ or AGR2+ BMI1+, and were more tolerant to treatment with the chemotherapeutic agents cisplatin and etoposide. Importantly, drug treatment of NG type cells resulted in the enrichment of CD133+ Sox2+ or AGR2+ BMI1+ cells.

Gene expression profiling has been employed to grade, identify and characterize various sub-types within tumors. In this study, unsupervised hierarchical clustering of tumor samples divided tumors into two groups that were primarily different in their stem cell pathways. In addition, three of the top differentially up-regulated genes Reelin, Calbindin 1 and Anterior Gradient 2 Homolog, and two of the most down differentially regulated genes Cytochrome P450 and F-Box Protein 32 are associated with stemness [37–41]. Unfortunately, it was not possible to analyze RNA for all tissues that generated cell lines due to lower achieved quality and often less available fresh tissue material for those tissues that generated cell lines. However, we compared RNA expression levels of AGR2 published by an independent study [42]. Their data from 68 patients support our findings as AGR2 expression reported in GEO showed a significantly higher average expression levels in grade (II+) tumors/or tumors that reoccurred compared to grade I tumours (grade I: 104.285, grade II+/reoccurred: 988.182, T test P 0.021). Importantly, tissue immunofluorescence used for four tumors, supported gene expression results for finding differences in stemness expression. In addition, CSCs rich areas appear to be also rich in AGR2 expression. AGR2 has been shown to be up regulated in breast, prostate and pancreatic cancers. Its function has been associated with the suppression of p53 phosphorylation and it was shown to interact with metastatic associated proteins [43]. However, its association with high grade meningioma is novel.

Most importantly, tumors that formed group 2, with low stem cell gene expression, generated G type cell lines that also had a lower expression of stem cell related markers, while tumors that showed high stem cell gene expression (including Jed45_MN) generated NG type cell lines that showed similar characteristics. This indicated that the generated primary cell lines do represent the essence of their corresponding tumors’ tissues, as previously shown with glioblastoma multiform tumors [44]. Thus functional characteristics observed in the retrieved cell lines are likely to benefit translational research for meningiomas. Unfortunately a few limitation of these primary cell lines remain, including full knowledge of the biochemical and genetic changes that they undergo from the point of initiation, difficulties encountered when attempting to harvest a large number of cells for end-point analysis while preserving enough cells for further culturing, as well as difficulties in establishing xenografts models, especially for the slow growing tumors [45]. Thus, further work is required to improve knowledge related to these features. In addition, it would be beneficial to increase the number of cell lines analysed in the future in order to verify analysis on a larger scale.

Clear biological features, including morphology which is influenced by the repository of biochemical interactions flowing through the cell signaling system [46], separated the derived meningioma cell lines into two groups. Pleomorphic features and multilayers were observed in previous studies and interpretations of these features led to a classification of slow or fast growing types [26, 27], however no association to stemness or drug resistance was made. The pleomorphic feature of NG type cell lines may be able to influence growth as cellular diversity could result in the production of a variety of growth factors that may promote proliferation. It is also possible that NG type cell lines have a high potential for cell division through selection upon retrieval of actively proliferating cells. It is worth noting that all of G type cell lines were derived from grade I tumors while four out of five NG type cell lines had come from tumor tissues observed to have a relatively high mitotic index count. It is also possible that the smaller bipolar neuronal-like cells may be mechanically more capable of division [47]. Notably, NG type untreated cell lines had significantly less nuclear Caspase-3, which may have enabled inhibition of apoptosis and promoted proliferation [48].

Previous work has shown the presence of cancer stem cells in meningioma cell lines [28–31], but did not associated novel markers with growth dynamic. In culture, both NG type and G type cell lines had cells co-positive for stem cell markers, however, NG cell lines showed increased number of CD133+ Sox2+ and AGR2+ BMI1+ co-positive cells. It is likely that the high frequencies of CSCs in NG cell lines are important for tumor dynamic growth and concurs with the notion that CSCs are pluripotent and generate pleomorphic cells which are likely to form pleomorphic tumors. However, it is important to consider that unlike stem cells, the precise cycling nature of cancer stem cells is still debatable and appears to be micro-environmentally influenced [49]. In addition, although the number of CD133+ Sox2+ cells was higher in NG type cell lines compared to the G type, an average of 34.6% of all NG type cells were positive, thus representing only a fraction of the total cell number, and could not necessary be solely accountable for the growth dynamic of this type of cell lines. Importantly, Nestin expression was relatively high in both types. However, Nestin positive cells that were not proliferating, were more frequently detected in G-type cells. As well as being detected in stem cells, Nestin expression has been reported in several differentiated cells including astrocytes, and its acknowledgement as an exclusive marker for CSCs is still debatable [50–52]. Perhaps in meningioma, Nestin overexpression occurs as an early event in the process of CSCs generation. This event could either be followed by further deregulations in stem cell-genes, such as Sox2, which result in the development of aggressive cancer stem cells, or if no further deregulations of stem cell-genes occur then a state of cell differentiation-like is encouraged instead. This is consistent with the hypothesis that CSCs develop through clonal evolution and become more complex as tumors progress [53]. Importantly, our data shows a novel association of AGR2 in meningioma stem cells. It is of interest to note that while staining Jed40_MN tissue revealed very few cells stained with AGR2, the corresponding cell line showed an average of 20% of cells that expressed AGR2. This suggests that for cell lines there might be a minimal threshold for AGR2 expression, perhaps to support survival under artificial/stressful conditions. Together, these results support a practice of stem-molecular staining for functional classification of high grade tumors and sub-typing of meningiomas.

Importantly, NG type cell lines showed a significantly higher tolerance of cisplatin and etoposide treatment compared with G type cell lines. Although etoposide has been traditionally thought of as a cell-cycle specific drug, recent data suggests a strong binding of the drug to chromatin and implicates a binding affinity for histone proteins, in particular histone 1, thus contributing to DNA damage regardless of the cell cycle status [54, 55]. Notably, although untreated NG type cell lines had less cells that express nuclear Caspase-3, the difference in expression levels between the G and NG type cell lines was maintained following treatment. This finding highlights a possible role of apoptosis avoidance and promotes the notion of nuclear staining of Caspase-3 as a useful biomarker of resistance or susceptibility to cisplatin and/or etoposide. However Caspase-3 requires further pre-clinical validation in appropriate animal models and proof-of-concept in human clinical trials for meningiomas. Importantly, CD133+ Sox2+, Nestin+ ki67+ and AGR2+ BMI1+ cells survived following cisplatin or etoposide treatment of NG type cells. The frequencies of surviving cells that express the three combinations of markers were different, suggesting that similar to glioblastoma, heterogeneous populations of CSCs may exist in meningioma [56]. Future experiments are required to investigate this possible diversity and the molecular basis of drug resistance by CSCs. A number of therapeutic strategies against CSCs are underway [57].

In conclusion, analysis of patterns for cellular architecture, drug tolerance and associated resistance makers for primary cell lines in combination with genomic analysis of corresponding tissues of meningiomas, led to the functional sub grouping of meningiomas and the identification of novel stem cells related markers that appear to be associated with drug resistance and are likely to play a role in tumor aggressiveness. Importantly, this work highlights the need to use stem cells molecular markers and early in vitro analysis to classify functional variants of meningioma and predicts tumor aggressiveness and drug resistance.

## Additional files

**Additional file 1: Figure S1.** Growth inhibition assays for A) Cisplatin and B) Etoposide for NG cell lines Jed38_MN, Jed45_MN and Jed49_MN.

**Additional file 2: Figure S2.** H&E slides for studied variants including meningothelial (Jed18_MN, Jed34_MN, and Jed39_MN) showing lobules of uniform eosinopholic cells with central nuclei and intranuclear inclusions forming abundant whorls with no evidence of atypia, necrosis or mitosis; fibroblastic (Jed33_MN, Jed40_MN, and Jed49_MN) showing spindle cells with indistinct cell boundaries running in fascicle; transitional (Jed04_MN, Jed36_MN, and Jed38_MN) with ratios of meningothelial to fibroblastic patterns ranging from 20:80 for Jed04_MN, 50:50 for Jed09_MN, 30:70 for Jed36_MN, and 40:60 for Jed38_MN; rhabdoid (Jed45_MN) showing hypercellular sheets with rhabdoid morphology (eccentric pleomorphic nuclei, abundant esinophilic cytoplasm) and necrosis; psammomatous (Jed43_MN) composed of whorled clusters of spindle cells with numerous psammoma bodies; and angiomatous (Jed12_MN) showing neoplastic growth in the form of nests and whorled of bland- looking polygonal cells with vascular component exceeding 50% of total tumor area and with no evidence of atypia, necrosis or mitosis. All images were taken at 20×.

**Additional file 3: Table S1.** Differentially expressed genes between group 1 (Tumors Jed49_MN, Jed36_MN) versus group 2 (Tumors Jed04_MN, Jed18_MN, Jed34_MN, Jed40_MN).

**Additional file 4: Table S2.** Predicted differential pathways generated by Ingenuity Pathway Analysis Software. The transcriptome comparison was carried out for group 1 (Tumors Jed49_MN, Jed36_MN) versus group 2 (Tumors Jed04_MN, Jed18_MN, Jed34_MN, Jed40_MN).

**Additional file 5: Figure S3.** CSCs markers expression in situ. A) Images for immunofluorescence co-staining of stem cell markers CD133+Sox2+ (Green, Red) or Nestin+Ki67+ (Green, Red) and AGR2+ BMI1+ (Red, Green) in low grade (Jed62_MN) and high grade (Jed45_MN) tumors. B) Mean percentages of co-positive cells. Error bars represent count errors between three independent regions within each tissue. All images were taken at 20×.

**Additional file 6: Figure S4.** AGR2 co-expression with CSCs markers in situ. A) Images for immunofluorescence co-staining of stem cell markers Nestin+AGR2+(Green, Red), CD133+AGR2+(Green, Red), and Sox2+AGR2+ (Green, Red) in low grade (Jed62_MN and Jed40_MN) and high grade (Jed49_MN and Jed45_MN) tumors. B) Mean percentages of co-positive cells. Error bars represent count errors between three independent regions within each tissue. All images were taken at 20×.

**Additional file 7: Figure S5.** Average percentages of morphologies counts for Jed62_MN and Jed79_MN. Each tumor was divided into four portions that were grown in either DMEM-F12 +10% FBS (Blue line), or DMEM high glucose concentrations of 4500 mg/L (Gibco) +10% FBS (Grey line), or DMEM high glucose concentrations of 4500 mg/L (Gibco) + 5% FBS (Yellow line), or DMEM low glucose concentrations of 1000 mg/L (Gibco) + 10% FBS (Orange line). Horizontal accesses represent morphologies described in Figure 2a (M: M Type, N: N Type, O:G: O Type, G: G Type, A: A Type, and D: D Type). A minimum of 500 cells were counted per week per cell line, per condition.

**Additional file 8: Figure S6.** The expression of AGR2 in G type cell lines. A) Immunofluorescence images for two G Type cell lines (Jed39_MN and Jed40_MN). Images show cells co-stained positively for Vimentin+AGR2+ (Green, Red). B) Average percentages of cells positive for AGR2. Error bars represent errors between three independent counts.

## Abbreviations

AGR2: anterior gradient 2 homolog
AKT1: RAC-alpha serine/threonine-protein kinase1
CALB1: calbindin 1
CNS: central nervous system tumors
CYP4B1: family 4 subfamily B polypeptide 1
FBXO32: F-box protein 32
GEP: gene expression profiling
H&E: hematoxylin and eosin stain
HBSS: Hank’s balanced salt solution
IPA: Ingenuity Pathways Analysis Software
KLF4: Kruppel-like factor
KLRC4: killer cell lectin-like receptor subfamily C, member 4
MEDAG: mesenteric estrogen-dependent adipogenesis
NF2: type 2 neurofibromatosis
PFA: paraformaldehyde
PIK3: phosphoinositide 3-kinase
RELN: reelin
SALL4: spalt-like transcription factor 4
SERPINA3: clade A (alpha-1 antiproteinase, antitrypsin), member 3
SMO: G protein-coupled receptor smoothened
TRAF7: TNF receptor-associated factor 7
WHO: World Health Organization
WNT6: wingless-type MMTV integration site family, member 6
