# Supplementary figures and images for: Pleomorphism and drug resistant cancer stem cells are characteristic of aggressive primary meningioma cell lines
Source: Cancer Cell Int. 2017 Jul 21;17:72. doi: 10.1186/s12935-017-0441-7 (PMC5521079; doi:10.1186/s12935-017-0441-7)

## Slide 1
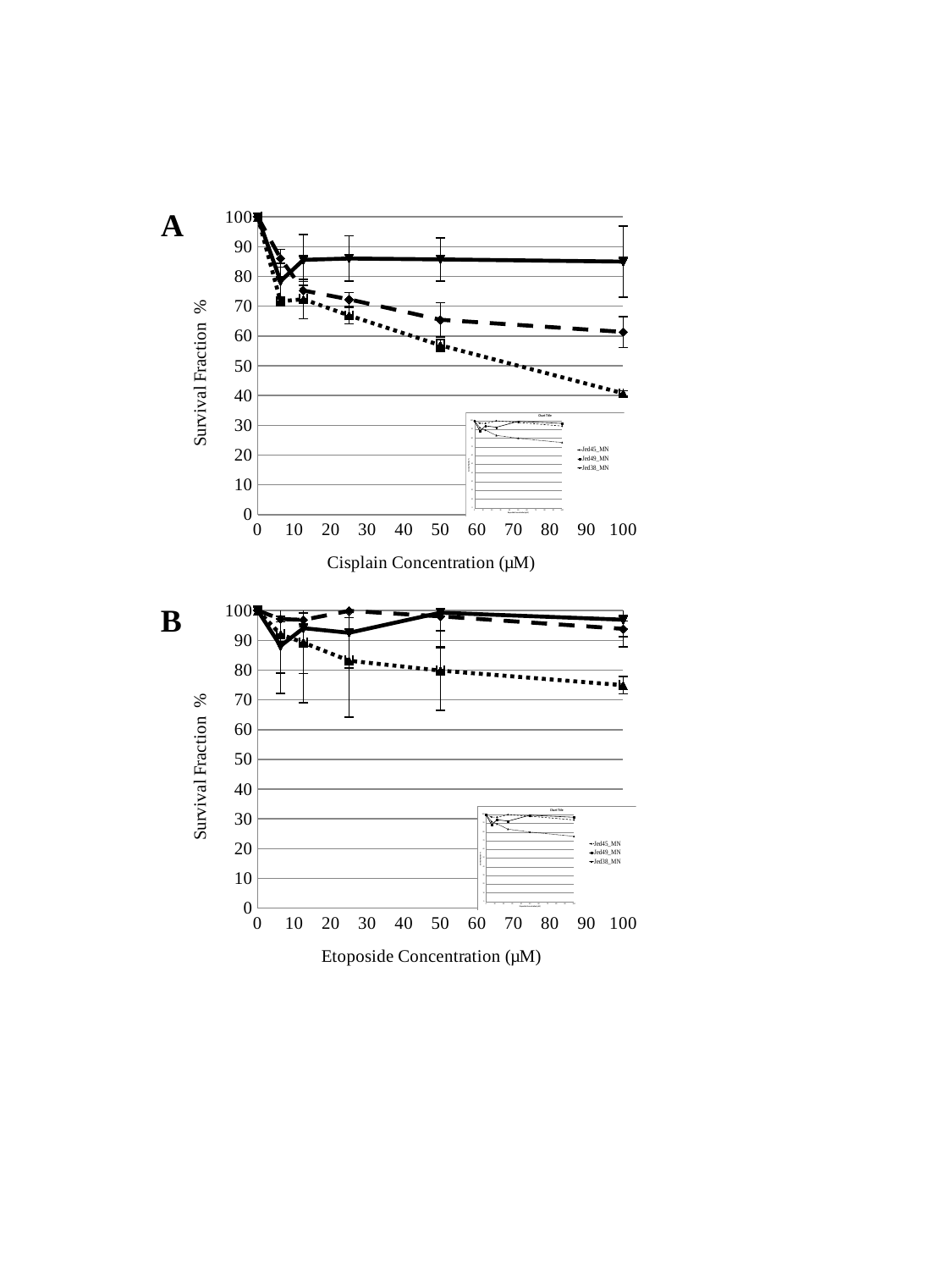

A
### Chart
| Category | Jed45_MN | Jed49_MN | Jed38_MN |
|---|---|---|---|
### Chart
| Category | Jed45_MN | Jed49_MN | Jed38_MN |
|---|---|---|---|B

Supplement: Supplementary file 1 — Additional file 1: Figure S1. Growth inhibition assays for A) Cisplatin and B) Etoposide for NG cell lines Jed38_MN, Jed45_MN and Jed49_MN. [file 12935_2017_441_MOESM1_ESM.pptx]

## Slide 1
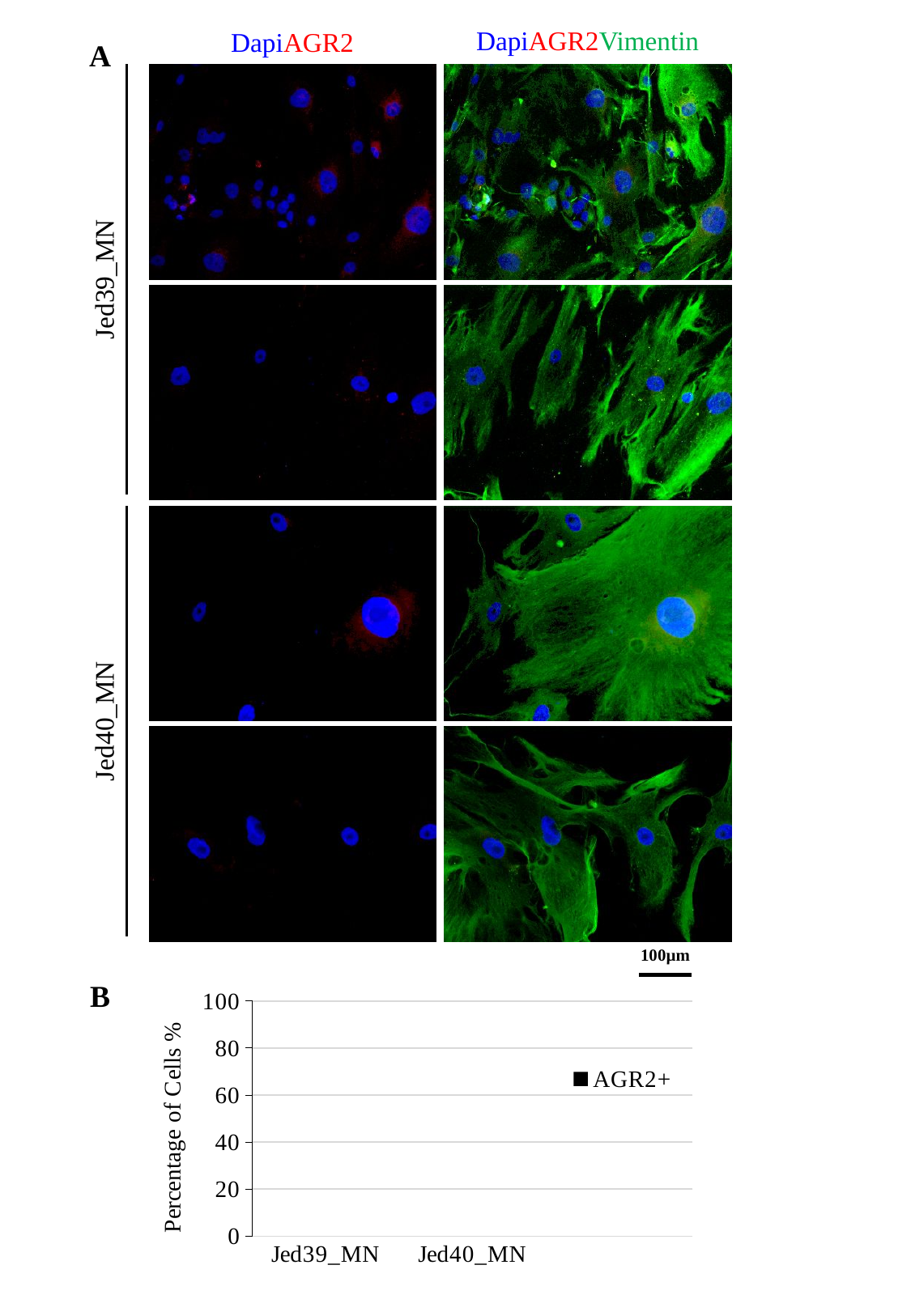

DapiAGR2Vimentin
DapiAGR2
A
Jed39_MN
Jed40_MN
100µm
B
### Chart
| Category | AGR2+ |
|---|---|
| Jed39_MN | 25.1043561697909 |
| Jed40_MN | 19.811790839828223 |

Supplement: Supplementary file 8 — Additional file 8: Figure S6. The expression of AGR2 in G type cell lines. A) Immunofluorescence images for two G Type cell lines (Jed39_MN and Jed40_MN). Images show cells co-stained positively for Vimentin+AGR2+ (Green, Red). B) Average percentages of cells positive for AGR2. Error bars represent errors between three independent counts. [file 12935_2017_441_MOESM8_ESM.pptx]
