# Supplementary material for: Pleomorphism and drug resistant cancer stem cells are characteristic of aggressive primary meningioma cell lines
Source: Cancer Cell Int. 2017 Jul 21;17:72. doi: 10.1186/s12935-017-0441-7 (PMC5521079; doi:10.1186/s12935-017-0441-7)
Supplement: Supplementary file 2 — Additional file 2: Figure S2. H&E slides for studied variants including meningothelial (Jed18_MN, Jed34_MN, and Jed39_MN) showing lobules of uniform eosinopholic cells with central nuclei and intranuclear inclusions forming abundant whorls with no evidence of atypia, necrosis or mitosis; fibroblastic (Jed33_MN, Jed40_MN, and Jed49_MN) showing spindle cells with indistinct cell boundaries running in fascicle; transitional (Jed04_MN, Jed36_MN, and Jed38_MN) with ratios of meningothelial to fibroblastic patterns ranging from 20:80 for Jed04_MN, 50:50 for Jed09_MN, 30:70 for Jed36_MN, and 40:60 for Jed38_MN; rhabdoid (Jed45_MN) showing hypercellular sheets with rhabdoid morphology (eccentric pleomorphic nuclei, abundant esinophilic cytoplasm) and necrosis; psammomatous (Jed43_MN) composed of whorled clusters of spindle cells with numerous psammoma bodies; and angiomatous (Jed12_MN) showing neoplastic growth in the form of nests and whorled of bland- looking polygonal cells with vascular component exceeding 50% of total tumor area and with no evidence of atypia, necrosis or mitosis. All images were taken at 20×. [file 12935_2017_441_MOESM2_ESM.ppt]

## Slide 1
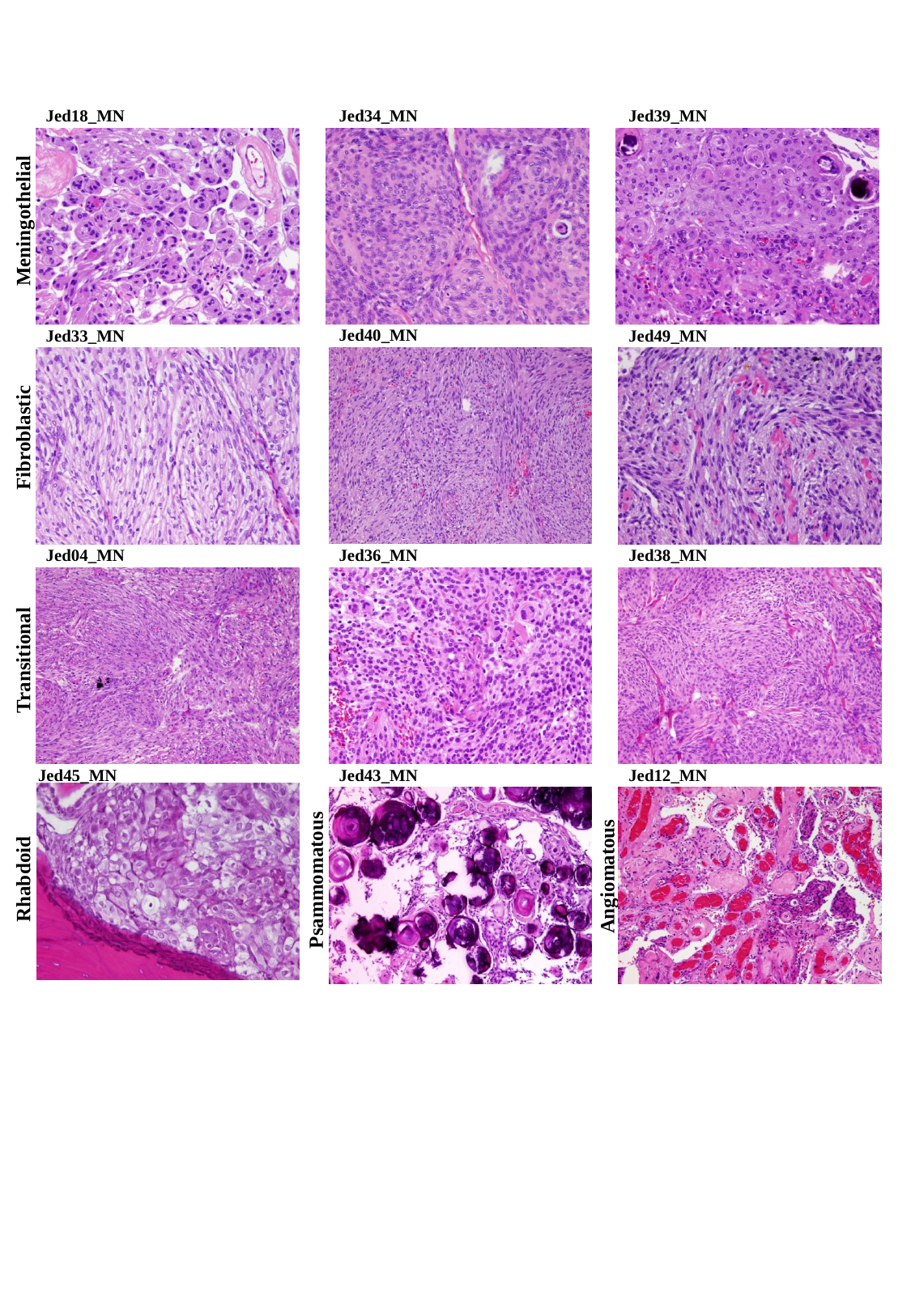

Jed18_MN
Jed34_MN
Jed39_MN
Meningothelial
Jed40_MN
Jed33_MN
Jed49_MN
Fibroblastic
Jed04_MN
Jed36_MN
Jed38_MN
Transitional
Jed45_MN
Jed43_MN
Jed12_MN
Angiomatous
Psammomatous
Rhabdoid
