# Supplementary material for: Pleomorphism and drug resistant cancer stem cells are characteristic of aggressive primary meningioma cell lines
Source: Cancer Cell Int. 2017 Jul 21;17:72. doi: 10.1186/s12935-017-0441-7 (PMC5521079; doi:10.1186/s12935-017-0441-7)
Supplement: Supplementary file 3 — Additional file 3: Table S1. Differentially expressed genes between group 1 (Tumors Jed49_MN, Jed36_MN) versus group 2 (Tumors Jed04_MN, Jed18_MN, Jed34_MN, Jed40_MN). [file 12935_2017_441_MOESM3_ESM.docx]

**Additional Table 1.** Differentially expressed genes between Group 1 (Tumours Jed49_MN, Jed36_MN) versus group 2 (Tumours Jed04_MN, Jed18_MN, Jed34_MN, Jed40_MN).

| **Gene Name** | **Gene Symbol** | **Bi-weight Avg Signal (log2)** | | **BD verses D** | |
| --- | --- | --- | --- | --- | --- |
|  |  | **BD** | **D** | **FC^1^** | **P** |
| **Up-Regulated Genes In BD** | | | | | |
| Reelin | RELN | 9.58 | 4.82 | 27.13 | 0.006998 |
| Calbindin 1, 28kda | CALB1 | 9.28 | 4.72 | 23.54 | 0.046713 |
| Killer Cell Lectin-Like Receptor Subfamily C, Member 4 | KLRC4 | 8.12 | 4.35 | 13.67 | 0.03339 |
| Anterior Gradient 2 Homolog (Xenopus Laevis) | AGR2 | 7.96 | 4.3 | 12.66 | 0.028752 |
| Neuropeptide Y Receptor Y6 (Pseudogene) | NPY6R | 8.78 | 5.21 | 11.89 | 0.034817 |
| Solute Carrier Family 22 (Organic Anion Transporter), Member 8 | SLC22A8 | 8.72 | 5.27 | 10.97 | 0.002309 |
| Klrc4-Klrk1 Readthrough; Killer Cell Lectin-Like Receptor Subfamily K, Member 1 | KLRC4-KLRK1; KLRK1 | 9.74 | 6.54 | 9.19 | 0.014318 |
| Neuregulin 1 | NRG1 | 7.96 | 4.99 | 7.85 | 0.003458 |
| Solute Carrier Family 16, Member 12 (Monocarboxylic Acid Transporter 12) | SLC16A12 | 9.09 | 6.23 | 7.24 | 0.03626 |
| Small Nucleolar Rna, C/D Box 115-20 | SNORD115-20 | 6.37 | 3.73 | 6.22 | 0.02471 |
| Small Nuclear Ribonucleoprotein Polypeptide N; Small Nucleolar Rna, C/D Box 115-5; Small Nucleolar Rna, C/D Box 115-9; Small Nucleolar Rna, C/D Box 115-11; Small Nucleolar Rna, C/D Box 115-12; Small Nucleolar Rna, C/D Box 115-22; Small Nucleolar Rna, C/D Box 115-26; Small Nucleolar Rna, C/D Box 115-29; Small Nucleolar Rna, C/D Box 115-36; Small Nucleolar Rna, C/D Box 115-43; Uncharacterized Loc100506948; Small Nucleolar Rna, C/D Box 116-28; Small Nucleolar Rna, C/D Box 115-13; Small Nucleolar Rna, C/D Box 115-7; Small Nucleolar Rna, C/D Box 107 | SNRPN; SNORD115-5; SNORD115-9; SNORD115-11; SNORD115-12; SNORD115-22; SNORD115-26; SNORD115-29; SNORD115-36; SNORD115-43; LOC100506948; SNORD116-28; SNORD115-13; SNORD115-7; SNORD107; SNHG14 | 7.64 | 5.08 | 5.91 | 0.020353 |
| Small Nuclear Ribonucleoprotein Polypeptide N; Small Nucleolar Rna, C/D Box 115-5; Small Nucleolar Rna, C/D Box 115-9; Small Nucleolar Rna, C/D Box 115-11; Small Nucleolar Rna, C/D Box 115-12; Small Nucleolar Rna, C/D Box 115-26; Small Nucleolar Rna, C/D Box 115-29; Small Nucleolar Rna, C/D Box 115-36; Small Nucleolar Rna, C/D Box 115-43; Uncharacterized Loc100506948; Small Nucleolar Rna, C/D Box 116-28; Small Nucleolar Rna, C/D Box 115-13; Small Nucleolar Rna, C/D Box 115-7; Small Nucleolar Rna, C/D Box 107 | SNRPN; SNORD115-5; SNORD115-9; SNORD115-11; SNORD115-12; SNORD115-26; SNORD115-29; SNORD115-36; SNORD115-43; LOC100506948; SNORD116-28; SNORD115-13; SNORD115-7; SNORD107; SNHG14 | 8.07 | 5.55 | 5.73 | 0.019369 |
| Small Nuclear Ribonucleoprotein Polypeptide N; Small Nucleolar Rna, C/D Box 115-5; Small Nucleolar Rna, C/D Box 115-9; Small Nucleolar Rna, C/D Box 115-11; Small Nucleolar Rna, C/D Box 115-12; Small Nucleolar Rna, C/D Box 115-26; Small Nucleolar Rna, C/D Box 115-29; Small Nucleolar Rna, C/D Box 115-36; Small Nucleolar Rna, C/D Box 115-43; Uncharacterized Loc100506948; Small Nucleolar Rna, C/D Box 116-28; Small Nucleolar Rna, C/D Box 115-13; Small Nucleolar Rna, C/D Box 115-7; Small Nucleolar Rna, C/D Box 107 | SNRPN; SNORD115-5; SNORD115-9; SNORD115-11; SNORD115-12; SNORD115-26; SNORD115-29; SNORD115-36; SNORD115-43; LOC100506948; SNORD116-28; SNORD115-13; SNORD115-7; SNORD107; SNHG14 | 8.05 | 5.54 | 5.72 | 0.020683 |
| Small Nuclear Ribonucleoprotein Polypeptide N; Small Nucleolar Rna, C/D Box 115-5; Small Nucleolar Rna, C/D Box 115-9; Small Nucleolar Rna, C/D Box 115-11; Small Nucleolar Rna, C/D Box 115-12; Small Nucleolar Rna, C/D Box 115-26; Small Nucleolar Rna, C/D Box 115-29; Small Nucleolar Rna, C/D Box 115-36; Small Nucleolar Rna, C/D Box 115-43; Uncharacterized Loc100506948; Small Nucleolar Rna, C/D Box 116-28; Small Nucleolar Rna, C/D Box 115-13; Small Nucleolar Rna, C/D Box 115-7; Small Nucleolar Rna, C/D Box 107 | SNRPN; SNORD115-5; SNORD115-9; SNORD115-11; SNORD115-12; SNORD115-26; SNORD115-29; SNORD115-36; SNORD115-43; LOC100506948; SNORD116-28; SNORD115-13; SNORD115-7; SNORD107; SNHG14 | 8.05 | 5.54 | 5.72 | 0.020683 |
| Small Nuclear Ribonucleoprotein Polypeptide N; Small Nucleolar Rna, C/D Box 115-5; Small Nucleolar Rna, C/D Box 115-9; Small Nucleolar Rna, C/D Box 115-11; Small Nucleolar Rna, C/D Box 115-12; Small Nucleolar Rna, C/D Box 115-26; Small Nucleolar Rna, C/D Box 115-29; Small Nucleolar Rna, C/D Box 115-36; Small Nucleolar Rna, C/D Box 115-43; Uncharacterized Loc100506948; Small Nucleolar Rna, C/D Box 116-28; Small Nucleolar Rna, C/D Box 115-13; Small Nucleolar Rna, C/D Box 115-7; Small Nucleolar Rna, C/D Box 107 | SNRPN; SNORD115-5; SNORD115-9; SNORD115-11; SNORD115-12; SNORD115-26; SNORD115-29; SNORD115-36; SNORD115-43; LOC100506948; SNORD116-28; SNORD115-13; SNORD115-7; SNORD107; SNHG14 | 8.05 | 5.54 | 5.72 | 0.020683 |
| Small Nuclear Ribonucleoprotein Polypeptide N; Small Nucleolar Rna, C/D Box 115-5; Small Nucleolar Rna, C/D Box 115-9; Small Nucleolar Rna, C/D Box 115-11; Small Nucleolar Rna, C/D Box 115-12; Small Nucleolar Rna, C/D Box 115-26; Small Nucleolar Rna, C/D Box 115-29; Small Nucleolar Rna, C/D Box 115-36; Small Nucleolar Rna, C/D Box 115-43; Uncharacterized Loc100506948; Small Nucleolar Rna, C/D Box 116-28; Small Nucleolar Rna, C/D Box 115-13; Small Nucleolar Rna, C/D Box 115-7; Small Nucleolar Rna, C/D Box 107 | SNRPN; SNORD115-5; SNORD115-9; SNORD115-11; SNORD115-12; SNORD115-26; SNORD115-29; SNORD115-36; SNORD115-43; LOC100506948; SNORD116-28; SNORD115-13; SNORD115-7; SNORD107; SNHG14 | 8.05 | 5.54 | 5.72 | 0.020683 |
| Klrc4-Klrk1 Readthrough; Killer Cell Lectin-Like Receptor Subfamily K, Member 1 | KLRC4-KLRK1; KLRK1 | 6.71 | 4.21 | 5.64 | 0.016503 |
| Small Nuclear Ribonucleoprotein Polypeptide N; Small Nucleolar Rna, C/D Box 115-5; Small Nucleolar Rna, C/D Box 115-9; Small Nucleolar Rna, C/D Box 115-11; Small Nucleolar Rna, C/D Box 115-12; Small Nucleolar Rna, C/D Box 115-26; Small Nucleolar Rna, C/D Box 115-29; Small Nucleolar Rna, C/D Box 115-36; Small Nucleolar Rna, C/D Box 115-43; Uncharacterized Loc100506948; Small Nucleolar Rna, C/D Box 116-28; Small Nucleolar Rna, C/D Box 115-13; Small Nucleolar Rna, C/D Box 115-7; Small Nucleolar Rna, C/D Box 107 | SNRPN; SNORD115-5; SNORD115-9; SNORD115-11; SNORD115-12; SNORD115-26; SNORD115-29; SNORD115-36; SNORD115-43; LOC100506948; SNORD116-28; SNORD115-13; SNORD115-7; SNORD107; SNHG14 | 7.96 | 5.49 | 5.56 | 0.022356 |
| Small Nuclear Ribonucleoprotein Polypeptide N; Small Nucleolar Rna, C/D Box 115-5; Small Nucleolar Rna, C/D Box 115-9; Small Nucleolar Rna, C/D Box 115-11; Small Nucleolar Rna, C/D Box 115-12; Small Nucleolar Rna, C/D Box 115-26; Small Nucleolar Rna, C/D Box 115-29; Small Nucleolar Rna, C/D Box 115-36; Small Nucleolar Rna, C/D Box 115-43; Uncharacterized Loc100506948; Small Nucleolar Rna, C/D Box 116-28; Small Nucleolar Rna, C/D Box 115-13; Small Nucleolar Rna, C/D Box 115-7; Small Nucleolar Rna, C/D Box 107 | SNRPN; SNORD115-5; SNORD115-9; SNORD115-11; SNORD115-12; SNORD115-26; SNORD115-29; SNORD115-36; SNORD115-43; LOC100506948; SNORD116-28; SNORD115-13; SNORD115-7; SNORD107; SNHG14 | 7.96 | 5.49 | 5.56 | 0.022356 |
| Small Nuclear Ribonucleoprotein Polypeptide N; Small Nucleolar Rna, C/D Box 115-5; Small Nucleolar Rna, C/D Box 115-9; Small Nucleolar Rna, C/D Box 115-11; Small Nucleolar Rna, C/D Box 115-12; Small Nucleolar Rna, C/D Box 115-26; Small Nucleolar Rna, C/D Box 115-29; Small Nucleolar Rna, C/D Box 115-36; Small Nucleolar Rna, C/D Box 115-43; Uncharacterized Loc100506948; Small Nucleolar Rna, C/D Box 116-28; Small Nucleolar Rna, C/D Box 115-13; Small Nucleolar Rna, C/D Box 115-7; Small Nucleolar Rna, C/D Box 107 | SNRPN; SNORD115-5; SNORD115-9; SNORD115-11; SNORD115-12; SNORD115-26; SNORD115-29; SNORD115-36; SNORD115-43; LOC100506948; SNORD116-28; SNORD115-13; SNORD115-7; SNORD107; SNHG14 | 7.96 | 5.49 | 5.56 | 0.022356 |
| Small Nuclear Ribonucleoprotein Polypeptide N; Small Nucleolar Rna, C/D Box 115-5; Small Nucleolar Rna, C/D Box 115-9; Small Nucleolar Rna, C/D Box 115-11; Small Nucleolar Rna, C/D Box 115-12; Small Nucleolar Rna, C/D Box 115-26; Small Nucleolar Rna, C/D Box 115-29; Small Nucleolar Rna, C/D Box 115-36; Small Nucleolar Rna, C/D Box 115-39; Small Nucleolar Rna, C/D Box 115-43; Uncharacterized Loc100506948; Small Nucleolar Rna, C/D Box 116-28; Small Nucleolar Rna, C/D Box 115-13; Small Nucleolar Rna, C/D Box 115-7; Small Nucleolar Rna, C/D Box 107 | SNRPN; SNORD115-5; SNORD115-9; SNORD115-11; SNORD115-12; SNORD115-26; SNORD115-29; SNORD115-36; SNORD115-39; SNORD115-43; LOC100506948; SNORD116-28; SNORD115-13; SNORD115-7; SNORD107; SNHG14 | 8.39 | 5.93 | 5.49 | 0.014237 |
| Ins-Igf2 Readthrough; Insulin-Like Growth Factor 2 (Somatomedin A) | INS-IGF2; IGF2 | 10.28 | 7.86 | 5.33 | 0.000824 |
| Thymocyte Selection-Associated High Mobility Group Box | TOX | 8.17 | 5.79 | 5.21 | 0.02492 |
| Synaptotagmin-Like 5 | SYTL5 | 6.3 | 3.93 | 5.17 | 0.024329 |
| Lipocalin-Like 1 | LCNL1 | 9.34 | 7.02 | 5.01 | 0.01462 |
| Histone Cluster 2, H2be | HIST2H2BE | 9.73 | 7.48 | 4.76 | 0.006849 |
| Rna, Ro-Associated Y1 Pseudogene 5 | RNY1P5 | 6.54 | 4.32 | 4.67 | 0.045352 |
| Testis Expressed 15 | TEX15 | 7.44 | 5.23 | 4.64 | 0.011169 |
| Syntrophin, Gamma 1 | SNTG1 | 6.05 | 3.85 | 4.6 | 0.027714 |
| Small Nucleolar Rna, C/D Box 115-17; Small Nucleolar Rna, C/D Box 115-18; Small Nucleolar Rna, C/D Box 115-19 | SNORD115-17; SNORD115-18; SNORD115-19 | 5.05 | 2.87 | 4.51 | 0.031667 |
| Small Nucleolar Rna, C/D Box 115-17; Small Nucleolar Rna, C/D Box 115-18; Small Nucleolar Rna, C/D Box 115-19 | SNORD115-17; SNORD115-18; SNORD115-19 | 5.05 | 2.87 | 4.51 | 0.031667 |
| Small Nucleolar Rna, C/D Box 115-17; Small Nucleolar Rna, C/D Box 115-18; Small Nucleolar Rna, C/D Box 115-19 | SNORD115-17; SNORD115-18; SNORD115-19 | 5.05 | 2.87 | 4.51 | 0.031667 |
| Long Intergenic Non-Protein Coding Rna 537; Rna, 5s Ribosomal Pseudogene 283; Rna, 5s Ribosomal Pseudogene 284 | LINC00537; RNA5SP283; RNA5SP284 | 8.89 | 6.78 | 4.3 | 0.030994 |
| Kallikrein B, Plasma (Fletcher Factor) 1 | KLKB1 | 7.36 | 5.3 | 4.17 | 0.046071 |
| Small Nucleolar Rna Host Gene 16 (Non-Protein Coding); Small Nucleolar Rna, C/D Box 1c | SNHG16; SNORD1C | 8.71 | 6.73 | 3.95 | 0.007307 |
| Pleckstrin Homology Domain Containing, Family G (With Rhogef Domain) Member 4b | PLEKHG4B | 8.67 | 6.73 | 3.84 | 0.028746 |
| Solute Carrier Family 38, Member 3 | SLC38A3 | 8.16 | 6.23 | 3.79 | 0.014245 |
| Coagulation Factor Xi | F11 | 7.69 | 5.82 | 3.67 | 0.012269 |
| Mitogen-Activated Protein Kinase 4 | MAPK4 | 8.29 | 6.41 | 3.67 | 0.024492 |
| Small Nucleolar Rna, C/D Box 115-6; Small Nucleolar Rna, C/D Box 115-42 | SNORD115-6; SNORD115-42 | 7.86 | 5.99 | 3.65 | 0.010529 |
| Hyaluronoglucosaminidase 1 | HYAL1 | 8.49 | 6.64 | 3.6 | 0.029396 |
| Sal-Like 4 (Drosophila) | SALL4 | 8.21 | 6.37 | 3.57 | 0.029676 |
| Small Nucleolar Rna, C/D Box 115-6; Small Nucleolar Rna, C/D Box 115-42 | SNORD115-6; SNORD115-42 | 7.62 | 5.83 | 3.47 | 0.010151 |
| Protein Kinase, Cgmp-Dependent, Type Ii | PRKG2 | 6.07 | 4.28 | 3.44 | 0.034186 |
| Growth Factor Receptor-Bound Protein 14 | GRB14 | 6.89 | 5.12 | 3.42 | 0.01986 |
| Troponin T Type 2 (Cardiac) | TNNT2 | 10.37 | 8.64 | 3.33 | 0.003471 |
| Microrna 181b-1 | MIR181B1 | 6.72 | 5.01 | 3.25 | 0.008451 |
| Small Nucleolar Rna, C/D Box 82 | SNORD82 | 9.05 | 7.35 | 3.25 | 0.042828 |
| Gamma-Aminobutyric Acid (Gaba) A Receptor, Alpha 3 | GABRA3 | 6.07 | 4.37 | 3.23 | 0.003174 |
| Olfactomedin 4 | OLFM4 | 6.81 | 5.14 | 3.19 | 0.016578 |
| Mannosidase, Endo-Alpha-Like | MANEAL | 8.2 | 6.53 | 3.17 | 0.000527 |
| Microrna 519a-1; Microrna 519a-2; Microrna 517c; Microrna 519e | MIR519A1; MIR519A2; MIR517C; MIR519E; CTC-339O9.1 | 3.9 | 2.25 | 3.16 | 0.034459 |
| Inositol-3-Phosphate Synthase 1 | ISYNA1 | 11.17 | 9.53 | 3.12 | 0.029517 |
| Solute Carrier Family 6 (Neurotransmitter Transporter, Gaba), Member 1 | SLC6A1 | 7.86 | 6.24 | 3.08 | 0.044727 |
| Immunoglobulin Heavy Constant Gamma 1 (G1m Marker); Single-Chain Fv Fragment; Immunoglobulin Heavy Constant Alpha 1; Immunoglobulin Heavy Constant Mu; Immunoglobulin Heavy Variable 3-48; Chromosome 7 Open Reading Frame 66; Immunoglobulin Heavy Variable 3-64; Immunoglobulin Heavy Variable 3-11 (Gene/Pseudogene); Immunoglobulin Heavy Variable 3-13; Immunoglobulin Heavy Variable 3-72; Immunoglobulin Heavy Variable 3-16 (Non-Functional); Immunoglobulin Heavy Variable 3-74; Immunoglobulin Heavy Variable 3-21; Immunoglobulin Heavy Variable 3-30; Immunoglobulin Heavy Variable 3/Or16-13 (Non-Functional); Immunoglobulin Heavy Variable 3-19 (Pseudogene); Immunoglobulin Heavy Variable 3-52 (Pseudogene); Immunoglobulin Heavy Variable 3-62 (Pseudogene) | IGHG1; SCFV; IGHA1; IGHM; IGHV3-48; C7orf66; IGHV3-64; IGHV3-11; IGHV3-13; IGHV3-72; IGHV3-16; IGHV3-74; IGHV3-21; IGHV3-30; IGHV3OR16-13; IGHV3-19; IGHV3-52; IGHV3-62; RP11-170L3.7; RP11-1166P10.5 | 9.73 | 8.11 | 3.07 | 0.048656 |
| Oxoglutarate Dehydrogenase-Like | OGDHL | 8.51 | 6.9 | 3.05 | 0.023116 |
| Small Nuclear Ribonucleoprotein Polypeptide N; Small Nucleolar Rna, C/D Box 115-1; Small Nucleolar Rna, C/D Box 115-13; Small Nucleolar Rna, C/D Box 115-16; Uncharacterized Loc100506948; Small Nucleolar Rna, C/D Box 116-28; Small Nucleolar Rna, C/D Box 115-26; Small Nucleolar Rna, C/D Box 115-7; Small Nucleolar Rna, C/D Box 107 | SNRPN; SNORD115-1; SNORD115-13; SNORD115-16; LOC100506948; SNORD116-28; SNORD115-26; SNORD115-7; SNORD107; SNHG14 | 7.45 | 5.85 | 3.04 | 0.00517 |
| Small Nuclear Ribonucleoprotein Polypeptide N; Small Nucleolar Rna, C/D Box 115-1; Small Nucleolar Rna, C/D Box 115-13; Small Nucleolar Rna, C/D Box 115-16; Uncharacterized Loc100506948; Small Nucleolar Rna, C/D Box 116-28; Small Nucleolar Rna, C/D Box 115-26; Small Nucleolar Rna, C/D Box 115-7; Small Nucleolar Rna, C/D Box 107 | SNRPN; SNORD115-1; SNORD115-13; SNORD115-16; LOC100506948; SNORD116-28; SNORD115-26; SNORD115-7; SNORD107; SNHG14 | 7.45 | 5.85 | 3.04 | 0.00517 |
| Small Nuclear Ribonucleoprotein Polypeptide N; Small Nucleolar Rna, C/D Box 115-1; Small Nucleolar Rna, C/D Box 115-13; Small Nucleolar Rna, C/D Box 115-16; Uncharacterized Loc100506948; Small Nucleolar Rna, C/D Box 116-28; Small Nucleolar Rna, C/D Box 115-26; Small Nucleolar Rna, C/D Box 115-7; Small Nucleolar Rna, C/D Box 107 | SNRPN; SNORD115-1; SNORD115-13; SNORD115-16; LOC100506948; SNORD116-28; SNORD115-26; SNORD115-7; SNORD107; SNHG14 | 7.45 | 5.85 | 3.04 | 0.00517 |
| Potassium Inwardly-Rectifying Channel, Subfamily J, Member 13 | KCNJ13 | 5.86 | 4.27 | 3.01 | 0.036182 |
| Small Nuclear Ribonucleoprotein Polypeptide N; Small Nucleolar Rna, C/D Box 115-7; Uncharacterized Loc100506948; Small Nucleolar Rna, C/D Box 116-28; Small Nucleolar Rna, C/D Box 115-26; Small Nucleolar Rna, C/D Box 115-13; Small Nucleolar Rna, C/D Box 107 | SNRPN; SNORD115-7; LOC100506948; SNORD116-28; SNORD115-26; SNORD115-13; SNORD107; SNHG14 | 4.64 | 3.07 | 2.97 | 0.001615 |
| Neuron Navigator 1 | NAV1 | 10.3 | 8.79 | 2.83 | 0.049508 |
| Solute Carrier Family 29 (Nucleoside Transporters), Member 4 | SLC29A4 | 7.92 | 6.45 | 2.78 | 0.005238 |
| Potassium Inwardly-Rectifying Channel, Subfamily J, Member 8 | KCNJ8 | 9.08 | 7.61 | 2.78 | 0.046995 |
| Rna, 5s Ribosomal Pseudogene 519 | RNA5SP519 | 7.31 | 5.84 | 2.77 | 0.021638 |
| Uncharacterized Loc100996689; Rna, 5s Ribosomal Pseudogene 283 | LOC100996689; RNA5SP283 | 9.89 | 8.44 | 2.73 | 0.040967 |
| Citrate Lyase Beta Like | CLYBL | 8.53 | 7.1 | 2.7 | 0.007238 |
| Small Nucleolar Rna, C/D Box 15a | SNORD15A | 8.55 | 7.13 | 2.67 | 0.001168 |
| Rho Gtpase Activating Protein 20 | ARHGAP20 | 9.89 | 8.48 | 2.66 | 0.021327 |
| Calponin Homology Domain Containing 2 | CHDC2; CXorf59 | 5.44 | 4.04 | 2.65 | 0.02188 |
| Transmembrane Protein 176a | TMEM176A | 8.17 | 6.77 | 2.65 | 0.026243 |
| 2,4-Dienoyl Coa Reductase 1, Mitochondrial | DECR1 | 9.68 | 8.28 | 2.64 | 0.002173 |
| Mesenchyme Homeobox 2 | MEOX2 | 11.16 | 9.76 | 2.64 | 0.035211 |
| Immunoglobulin Heavy Variable 3-20; Immunoglobulin Heavy Variable 3-43 | IGHV3-20; IGHV3-43 | 9.67 | 8.28 | 2.61 | 0.036661 |
| Gamma-Aminobutyric Acid (Gaba) A Receptor, Theta | GABRQ | 5.99 | 4.6 | 2.61 | 0.036841 |
| Rna, Variant U1 Small Nuclear 13 | RNVU1-13 | 8.12 | 6.74 | 2.6 | 0.021913 |
| Cytochrome C Oxidase Subunit Vic | COX6C | 9.48 | 8.1 | 2.6 | 0.025536 |
| Ribosomal Protein S21 | RPS21 | 8.14 | 6.78 | 2.58 | 0.031092 |
| Small Nucleolar Rna, C/D Box 115-44 | SNORD115-44 | 6.06 | 4.71 | 2.55 | 0.006392 |
| Dehydrogenase/Reductase (Sdr Family) Member 11 | DHRS11 | 8.95 | 7.6 | 2.54 | 0.006394 |
| Dehydrogenase/Reductase (Sdr Family) Member 2 | DHRS2 | 6.4 | 5.07 | 2.52 | 0.006226 |
| Immunoglobulin Heavy Variable 3-72 | IGHV3-72 | 9.13 | 7.79 | 2.52 | 0.025875 |
| Aryl-Hydrocarbon Receptor Nuclear Translocator 2 | ARNT2 | 10.45 | 9.14 | 2.49 | 0.006403 |
| Ribosomal Protein L13a; Small Nucleolar RNA, C/D Box 35A; Small Nucleolar RNA, C/D Box 32A; Small Nucleolar RNA, C/D Box 33; Small Nucleolar RNA, C/D Box 34 | RPL13A; SNORD35A; SNORD32A; SNORD33; SNORD34 | 10.97 | 9.65 | 2.49 | 0.036903 |
| Small Nucleolar Rna, H/Aca Box 71c | SNORA71C | 9.18 | 7.88 | 2.47 | 0.025336 |
| Small Nucleolar Rna, C/D Box 115-41 | SNORD115-41 | 3.54 | 2.24 | 2.46 | 0.021117 |
| Wnt1 Inducible Signaling Pathway Protein 1 | WISP1 | 7.78 | 6.49 | 2.44 | 0.009891 |
| Small Nucleolar Rna, C/D Box 13 Pseudogene 1 | SNORD13P1 | 4.24 | 2.95 | 2.44 | 0.036054 |
| Sialic Acid Acetylesterase | SIAE | 9.6 | 8.32 | 2.43 | 0.017928 |
| Chemokine (C-C Motif) Receptor 9 | CCR9 | 6.63 | 5.35 | 2.43 | 0.034315 |
| Small Nucleolar Rna, C/D Box 115-25 | SNORD115-25 | 5.39 | 4.11 | 2.42 | 0.007453 |
| Small Nucleolar Rna, C/D Box 42b | SNORD42B | 7.12 | 5.86 | 2.38 | 0.011753 |
| Wingless-Type Mmtv Integration Site Family, Member 6 | WNT6 | 8.28 | 7.05 | 2.34 | 0.003313 |
| Folate Hydrolase 1b; Folate Hydrolase (Prostate-Specific Membrane Antigen) 1 | FOLH1B; FOLH1 | 5.18 | 3.96 | 2.33 | 0.028537 |
| Glutamic-Oxaloacetic Transaminase 1, Soluble | GOT1 | 10.6 | 9.38 | 2.32 | 0.045441 |
| G Protein-Coupled Receptor 1 | GPR1 | 5.39 | 4.18 | 2.31 | 0.019016 |
| Long Intergenic Non-Protein Coding Rna 189 | LINC00189 | 6.26 | 5.06 | 2.3 | 0.004699 |
| Rna, 5s Ribosomal Pseudogene 398 | RNA5SP398 | 6.44 | 5.24 | 2.29 | 0.012307 |
| Purkinje Cell Protein 4 | PCP4 | 6.31 | 5.12 | 2.28 | 0.039829 |
| Aldo-Keto Reductase Family 1, Member E2 | AKR1E2 | 6.73 | 5.55 | 2.26 | 0.010022 |
| Ribosomal Protein L10; Small Nucleolar Rna, H/Aca Box 70 | RPL10; SNORA70 | 11.83 | 10.66 | 2.24 | 0.0056 |
| Small Nucleolar Rna, H/Aca Box 48; Small Nucleolar Rna, C/D Box 10; Small Nucleolar Rna, H/Aca Box 67; Eukaryotic Translation Initiation Factor 4a1 | SNORA48; SNORD10; SNORA67; EIF4A1 | 10.37 | 9.21 | 2.23 | 0.018785 |
| Uncharacterized LOC646903 | LOC646903 | 6.34 | 5.18 | 2.23 | 0.047007 |
| Pleckstrin Homology Domain Containing, Family G (With Rhogef Domain) Member 2 | PLEKHG2 | 8.28 | 7.14 | 2.21 | 0.017547 |
| Chemokine (C-X-C Motif) Ligand 12 | CXCL12 | 8.25 | 7.13 | 2.18 | 0.006528 |
| Small Nucleolar Rna, C/D Box 115-6; Small Nucleolar Rna, C/D Box 115-30; Small Nucleolar Rna, C/D Box 115-42 | SNORD115-6; SNORD115-30; SNORD115-42 | 3.7 | 2.58 | 2.18 | 0.01613 |
| Uncharacterized LOC100131860 | LOC100131860 | 7.65 | 6.53 | 2.17 | 0.034431 |
| NADH Dehydrogenase (Ubiquinone) 1 Alpha Subcomplex, 1, 7.5kda | NDUFA1 | 10.09 | 8.98 | 2.16 | 0.006178 |
| Folate Hydrolase (Prostate-Specific Membrane Antigen) 1; Folate Hydrolase 1b | FOLH1; FOLH1B | 5.2 | 4.09 | 2.16 | 0.027738 |
| Tetraspanin 13 | TSPAN13 | 6.96 | 5.85 | 2.15 | 0.010435 |
| Carbonic Anhydrase Ii | CA2 | 6.87 | 5.77 | 2.15 | 0.011356 |
| Immunoglobulin Heavy Variable 3-48; Immunoglobulin Heavy Variable 3-53; Immunoglobulin Heavy Variable 3-66 | IGHV3-48; IGHV3-53; IGHV3-66 | 9.79 | 8.68 | 2.15 | 0.02945 |
| Coiled-Coil-Helix-Coiled-Coil-Helix Domain Containing 10 | CHCHD10 | 8.73 | 7.63 | 2.14 | 0.005749 |
| Nfkb Inhibitor Interacting Ras-Like 2 | NKIRAS2 | 9.15 | 8.05 | 2.14 | 0.035067 |
| Small Nucleolar Rna, C/D Box 95; Small Nucleolar Rna, C/D Box 96a; Guanine Nucleotide Binding Protein (G Protein), Beta Polypeptide 2-Like 1 | SNORD95; SNORD96A; GNB2L1 | 10.66 | 9.57 | 2.14 | 0.037295 |
| Cd207 Molecule, Langerin | CD207 | 5.84 | 4.75 | 2.13 | 0.004068 |
| Uncharacterized Loc100506303; Uncharacterized Loc440157; Uncharacterized Loc400879 | LOC100506303; LOC440157; LOC400879; AL589743.1 | 6.34 | 5.25 | 2.13 | 0.008291 |
| Chromosome X Open Reading Frame 22 | CXorf22 | 5.46 | 4.37 | 2.13 | 0.014653 |
| Egf-Like Module Containing, Mucin-Like, Hormone Receptor-Like 1 | EMR1 | 5.92 | 4.84 | 2.12 | 0.02273 |
| Chromosome 7 Open Reading Frame 13 | C7orf13 | 6.64 | 5.55 | 2.12 | 0.024165 |
| Rna Binding Motif Protein 46 | RBM46 | 4.26 | 3.17 | 2.12 | 0.028807 |
| Small Nucleolar Rna, C/D Box 59a | SNORD59A | 5.24 | 4.15 | 2.12 | 0.032887 |
| Interleukin 2 Receptor, Alpha | IL2RA | 6.91 | 5.83 | 2.12 | 0.039619 |
| Mir4697 Host Gene (Non-Protein Coding) | MIR4697HG; RP11-259P6.2 | 6.61 | 5.54 | 2.11 | 0.025887 |
| NADH Dehydrogenase (Ubiquinone) 1 Alpha Subcomplex, 2, 8kda | NDUFA2 | 9.75 | 8.69 | 2.09 | 0.013738 |
| Olfactory Receptor, Family 4, Subfamily C, Member 2 Pseudogene | OR4C2P | 4.04 | 2.97 | 2.09 | 0.021255 |
| D Site Of Albumin Promoter (Albumin D-Box) Binding Protein | DBP | 9.07 | 8.01 | 2.07 | 0.000207 |
| Zinc Finger, B-Box Domain Containing | ZBBX | 4.52 | 3.47 | 2.07 | 0.005094 |
| Rna, 5s Ribosomal Pseudogene 69 | RNA5SP69 | 6.99 | 5.94 | 2.07 | 0.009293 |
| Bpi Fold Containing Family A, Member 2 | BPIFA2 | 5.82 | 4.77 | 2.07 | 0.014884 |
| Hig1 Hypoxia Inducible Domain Family, Member 2a | HIGD2A | 11.56 | 10.52 | 2.07 | 0.021334 |
| Fibulin 7 | FBLN7 | 7.45 | 6.41 | 2.06 | 0.007404 |
| Immunoglobulin Heavy Constant Gamma 1 (G1m Marker); Immunoglobulin Heavy Variable 3-48; Immunoglobulin Heavy Variable 3-53; Immunoglobulin Heavy Variable 3-66; Immunoglobulin Heavy Variable 3-7; Immunoglobulin Heavy Variable 3-72; Immunoglobulin Heavy Variable 3-21; Immunoglobulin Heavy Variable 3-38 (Non-Functional) | IGHG1; IGHV3-48; IGHV3-53; IGHV3-66; IGHV3-7; IGHV3-72; IGHV3-21; IGHV3-38 | 10.36 | 9.32 | 2.06 | 0.028688 |
| Chromosome 19 Open Reading Frame 54 | C19orf54 | 8.54 | 7.5 | 2.05 | 0.001125 |
| Solute Carrier Family 6, Member 16 | SLC6A16 | 6.47 | 5.44 | 2.05 | 0.008587 |
| Nuclear Rna Export Factor 3 | NXF3 | 5.52 | 4.49 | 2.05 | 0.01655 |
| Membrane-Spanning 4-Domains, Subfamily A, Member 8 | MS4A8; MS4A8B | 5.88 | 4.85 | 2.03 | 0.003466 |
| Killer Cell Lectin-Like Receptor Subfamily C, Member 2 | KLRC2 | 3.69 | 2.67 | 2.03 | 0.009637 |
| Sialic Acid Binding Ig-Like Lectin 8 | SIGLEC8 | 7.61 | 6.59 | 2.03 | 0.027913 |
| Maestro | MRO | 7.03 | 6.01 | 2.03 | 0.034584 |
| Stearoyl-Coa Desaturase (Delta-9-Desaturase) | SCD | 5.16 | 4.14 | 2.03 | 0.04251 |
| T-Cell Leukemia/Lymphoma 6 (Non-Protein Coding) | TCL6 | 5.26 | 4.23 | 2.03 | 0.049224 |
| Uncharacterized LOC100132800 | LOC100132800; AC018755.15 | 6.58 | 5.58 | 2.01 | 0.007375 |
| Chromosome 10 Open Reading Frame 11 | C10orf11 | 8.32 | 7.31 | 2.01 | 0.008482 |
| NADH Dehydrogenase (Ubiquinone) 1 Beta Subcomplex, 5, 16kda | NDUFB5 | 10.87 | 9.87 | 2.01 | 0.023512 |
| **Down-Regulated Genes In BD** | | | | | |
| Cytochrome P450, Family 4, Subfamily B, Polypeptide 1 | CYP4B1 | 6.15 | 10.27 | -17.4 | 0.004088 |
| F-Box Protein 32 | FBXO32 | 6.17 | 10.17 | -15.98 | 0.013618 |
| Mesenteric Estrogen-Dependent Adipogenesis | MEDAG | 6.39 | 9.84 | -10.94 | 0.01099 |
| Serpin Peptidase Inhibitor, Clade A (Alpha-1 Antiproteinase, Antitrypsin), Member 3 | SERPINA3 | 5.47 | 8.63 | -8.95 | 0.038429 |
| Microfibrillar Associated Protein 5 | MFAP5 | 8.98 | 12.05 | -8.38 | 0.032452 |
| Ubiquitin Carboxyl-Terminal Esterase L1 (Ubiquitin Thiolesterase) | UCHL1 | 6.83 | 9.87 | -8.25 | 0.016931 |
| Leucine Rich Repeat Neuronal 1 | LRRN1 | 5.25 | 8.3 | -8.25 | 0.0003 |
| Paired Related Homeobox 1 | PRRX1 | 7.63 | 10.64 | -8.04 | 0.007537 |
| Latrophilin 2 | LPHN2 | 6.51 | 9.51 | -8.02 | 0.007481 |
| Potassium Large Conductance Calcium-Activated Channel, Subfamily M, Alpha Member 1 | KCNMA1 | 8.45 | 11.45 | -8.01 | 0.005359 |
| TIMP Metallopeptidase Inhibitor 3 | TIMP3 | 7.84 | 10.73 | -7.42 | 0.016338 |
| AF4/FMR2 Family, Member 2 | AFF2 | 6.73 | 9.61 | -7.37 | 0.005402 |
| Proteolipid Protein 1 | PLP1 | 6.67 | 9.5 | -7.11 | 0.032656 |
| Podocan | PODN | 7.09 | 9.91 | -7.05 | 0.00321 |
| Ethanolamine Kinase 2 | ETNK2 | 5.64 | 8.43 | -6.91 | 0.003478 |
| Heat Shock 22kda Protein 8 | HSPB8 | 5.52 | 8.26 | -6.7 | 0.001244 |
| Synaptopodin 2 | SYNPO2 | 7.29 | 10.01 | -6.61 | 0.026885 |
| Myosin Light Chain Kinase Family, Member 4 | MYLK4 | 5.9 | 8.45 | -5.86 | 0.026751 |
| Melanocortin 2 Receptor Accessory Protein 2 | MRAP2 | 5.92 | 8.44 | -5.72 | 0.015929 |
| Glutathione S-Transferase Mu 3 (Brain) | GSTM3 | 6.69 | 9.19 | -5.66 | 0.019052 |
| Latent Transforming Growth Factor Beta Binding Protein 1 | LTBP1 | 6.7 | 9.17 | -5.53 | 0.014895 |
| T-Box 18 | TBX18 | 6.4 | 8.81 | -5.34 | 0.001035 |
| Myosin VB | MYO5B | 6.74 | 9.12 | -5.2 | 0.037781 |
| Insulin-Like Growth Factor Binding Protein 6 | IGFBP6 | 8.6 | 10.97 | -5.16 | 0.032975 |
| Phosphatidic Acid Phosphatase Type 2B | PPAP2B | 9.16 | 11.52 | -5.11 | 0.047679 |
| Glycoprotein (Transmembrane) Nmb | GPNMB | 8.11 | 10.44 | -5.02 | 0.019613 |
| Sarcoglycan, Epsilon | SGCE | 6.35 | 8.65 | -4.92 | 0.021509 |
| UDP Glycosyltransferase 3 Family, Polypeptide A2 | UGT3A2 | 7.55 | 9.82 | -4.8 | 0.048075 |
| Milk Fat Globule-EGF Factor 8 Protein | MFGE8 | 7.89 | 10.15 | -4.78 | 0.008498 |
| Transforming Growth Factor, Beta 3 | TGFB3 | 6.16 | 8.4 | -4.72 | 0.006247 |
| Cell Adhesion Associated, Oncogene Regulated | CDON | 7.62 | 9.8 | -4.54 | 0.014556 |
| Cadherin 3, Type 1, P-Cadherin (Placental) | CDH3 | 7.03 | 9.21 | -4.53 | 0.020535 |
| Retinol Binding Protein 4, Plasma | RBP4 | 5.11 | 7.24 | -4.37 | 0.024015 |
| Leucine Rich Repeat Containing 16A | LRRC16A | 6.38 | 8.48 | -4.29 | 0.021793 |
| Serpin Peptidase Inhibitor, Clade F (Alpha-2 Antiplasmin, Pigment Epithelium Derived Factor), Member 1 | SERPINF1 | 9.42 | 11.49 | -4.18 | 0.028646 |
| Androgen Receptor | AR | 6.3 | 8.33 | -4.1 | 0.028987 |
| Integrin, Alpha 11 | ITGA11 | 7.43 | 9.41 | -3.95 | 0.021865 |
| Ribosomal Protein S6 Kinase, 90kda, Polypeptide 2 | RPS6KA2 | 6.9 | 8.88 | -3.93 | 0.03961 |
| Mitogen-Activated Protein Kinase Kinase Kinase 5 | MAP3K5 | 6.76 | 8.72 | -3.9 | 0.025873 |
| Peptidase M20 Domain Containing 2 | PM20D2; RP11-63L7.4 | 6.53 | 8.49 | -3.89 | 0.008201 |
| Solute Carrier Family 16, Member 9 (Monocarboxylic Acid Transporter 9) | SLC16A9 | 6.32 | 8.21 | -3.71 | 0.029326 |
| SEC14-Like 2 (S. Cerevisiae) | SEC14L2 | 6.11 | 8 | -3.69 | 0.013617 |
| FYN Oncogene Related To SRC, FGR, YES | FYN | 6.77 | 8.62 | -3.61 | 0.03341 |
| Very Low Density Lipoprotein Receptor | VLDLR | 6.53 | 8.34 | -3.52 | 0.025511 |
| Solute Carrier Family 1 (Glutamate/Neutral Amino Acid Transporter), Member 4 | SLC1A4 | 7.52 | 9.34 | -3.52 | 0.035609 |
| Atlastin Gtpase 1 | ATL1 | 7.12 | 8.93 | -3.49 | 0.012791 |
| Transcription Elongation Factor A (SII)-Like 2 | TCEAL2 | 4.92 | 6.69 | -3.42 | 0.003894 |
| TIMP Metallopeptidase Inhibitor 3 | TIMP3 | 10.34 | 12.11 | -3.41 | 0.036314 |
| Ferric-Chelate Reductase 1-Like | FRRS1L | 7.29 | 9.05 | -3.39 | 0.032834 |
| Atlastin Gtpase 3 | ATL3 | 8.26 | 10.01 | -3.34 | 0.027669 |
| Peptidylprolyl Isomerase (Cyclophilin)-Like 4 | PPIL4 | 6.47 | 8.21 | -3.33 | 0.027221 |
| Insulin-Like Growth Factor Binding Protein 5 | IGFBP5 | 11.68 | 13.4 | -3.3 | 0.00981 |
| Rho Guanine Nucleotide Exchange Factor (GEF) 26 | ARHGEF26 | 5.11 | 6.82 | -3.29 | 0.013931 |
| Human Immunodeficiency Virus Type I Enhancer Binding Protein 2 | HIVEP2 | 6.38 | 8.09 | -3.28 | 0.005602 |
| ADAMTS-Like 3 | ADAMTSL3 | 9.29 | 10.95 | -3.16 | 0.006622 |
| Histidine Triad Nucleotide Binding Protein 3 | HINT3 | 5.7 | 7.34 | -3.13 | 0.037057 |
| Adenosylhomocysteinase-Like 2 | AHCYL2 | 10.26 | 11.89 | -3.1 | 0.02077 |
| Myosin ID | MYO1D | 6.59 | 8.22 | -3.09 | 0.040338 |
| Death-Associated Protein Kinase 1 | DAPK1 | 8.42 | 10.01 | -3.02 | 0.02101 |
| SUMO1/Sentrin Specific Peptidase 6 | SENP6 | 7.38 | 8.94 | -2.96 | 0.043843 |
| Gamma-Aminobutyric Acid (GABA) A Receptor, Beta 3 | GABRB3 | 6.32 | 7.88 | -2.95 | 0.01801 |
| Vacuolar Protein Sorting 4 Homolog B (S. Cerevisiae) | VPS4B | 6.92 | 8.46 | -2.91 | 0.049431 |
| Pleiomorphic Adenoma Gene-Like 1 | PLAGL1 | 6.74 | 8.27 | -2.88 | 0.019412 |
| Utrophin | UTRN | 7.95 | 9.47 | -2.86 | 0.032182 |
| Family With Sequence Similarity 229, Member B | FAM229B; C6orf225 | 6.49 | 7.99 | -2.83 | 0.015756 |
| Zinc Finger, C2HC-Type Containing 1A | ZC2HC1A | 6.67 | 8.17 | -2.82 | 0.0029 |
| Human Immunodeficiency Virus Type I Enhancer Binding Protein 1 | HIVEP1 | 7.3 | 8.78 | -2.79 | 0.0234 |
| Sterile Alpha Motif Domain Containing 12 | SAMD12 | 5.38 | 6.86 | -2.78 | 0.006722 |
| Protein Kinase, AMP-Activated, Alpha 2 Catalytic Subunit | PRKAA2 | 6.23 | 7.69 | -2.76 | 0.025081 |
| Solute Carrier Family 12 (Sodium/Potassium/Chloride Transporters), Member 2 | SLC12A2 | 8.73 | 10.18 | -2.74 | 0.047764 |
| REV3-Like, Polymerase (DNA Directed), Zeta, Catalytic Subunit | REV3L | 6.64 | 8.09 | -2.74 | 0.040202 |
| Dopey Family Member 1 | DOPEY1 | 6.54 | 8 | -2.74 | 0.02647 |
| Dedicator Of Cytokinesis 9 | DOCK9 | 7.8 | 9.25 | -2.72 | 0.019915 |
| Frizzled Family Receptor 3 | FZD3 | 6.36 | 7.79 | -2.68 | 0.006775 |
| RWD Domain Containing 4 | RWDD4 | 7.66 | 9.08 | -2.67 | 0.023996 |
| EF-Hand Calcium Binding Domain 14 | EFCAB14; KIAA0494 | 9.32 | 10.74 | -2.67 | 0.00673 |
| Calmodulin-Like 4 | CALML4 | 7.36 | 8.77 | -2.67 | 0.011077 |
| WD Repeat Domain 7 | WDR7 | 7.41 | 8.82 | -2.66 | 0.017196 |
| UDP-Gal:Betaglcnac Beta 1,4- Galactosyltransferase, Polypeptide 4 | B4GALT4 | 6.77 | 8.19 | -2.66 | 0.01563 |
| Tropomodulin 2 (Neuronal) | TMOD2 | 7.67 | 9.08 | -2.66 | 0.00987 |
| Taste Receptor, Type 2, Member 20 | TAS2R20 | 5.04 | 6.45 | -2.66 | 0.03174 |
| Heat Shock 105kda/110kda Protein 1 | HSPH1 | 8.05 | 9.46 | -2.66 | 0.011451 |
| Extended Synaptotagmin-Like Protein 3 | ESYT3 | 5.31 | 6.72 | -2.66 | 0.024233 |
| Dermatan Sulfate Epimerase-Like | DSEL | 5.83 | 7.23 | -2.65 | 0.01177 |
| Phospholipase C-Like 2 | PLCL2 | 5.87 | 7.27 | -2.64 | 0.040754 |
| Vesicle Amine Transport Protein 1 Homolog (T. Californica) | VAT1 | 8.81 | 10.21 | -2.63 | 0.016747 |
| CD200 Molecule | CD200 | 6.06 | 7.45 | -2.62 | 0.040628 |
| UFM1-Specific Ligase 1 | UFL1 | 7.8 | 9.19 | -2.61 | 0.040673 |
| Kiaa1107 | KIAA1107 | 5.74 | 7.12 | -2.61 | 0.005783 |
| HBS1-Like (S. Cerevisiae) | HBS1L | 7.25 | 8.64 | -2.61 | 0.007464 |
| CDC42 Effector Protein (Rho Gtpase Binding) 3 | CDC42EP3 | 7.68 | 9.07 | -2.61 | 0.030426 |
| Serine/Threonine Kinase 33 | STK33 | 5.31 | 6.68 | -2.58 | 0.001322 |
| Ras Homolog Family Member U | RHOU | 9.83 | 11.19 | -2.57 | 0.03754 |
| Chromosome 16 Open Reading Frame 45 | C16orf45 | 6.03 | 7.39 | -2.57 | 0.004092 |
| PR Domain Containing 6 | PRDM6 | 8.04 | 9.4 | -2.56 | 0.02391 |
| Nephroblastoma Overexpressed | NOV | 9.8 | 11.15 | -2.56 | 0.04241 |
| Microtubule-Associated Protein 7 | MAP7 | 5.96 | 7.32 | -2.56 | 0.002196 |
| Zinc Finger And BTB Domain Containing 21 | ZBTB21; ZNF295 | 6.98 | 8.32 | -2.53 | 0.025529 |
| Spondin 1, Extracellular Matrix Protein | SPON1 | 5.6 | 6.94 | -2.53 | 0.045313 |
| Serpin Peptidase Inhibitor, Clade B (Ovalbumin), Member 8 | SERPINB8 | 6.42 | 7.76 | -2.53 | 0.025438 |
| Stathmin 1 | STMN1 | 7.21 | 8.54 | -2.52 | 0.038939 |
| Mannosidase, Alpha, Class 1C, Member 1 | MAN1C1 | 8.38 | 9.71 | -2.51 | 0.022196 |
| RWD Domain Containing 4 | RWDD4 | 8.26 | 9.58 | -2.5 | 0.013143 |
| Katanin P60 Subunit A-Like 1 | KATNAL1 | 5.49 | 6.8 | -2.48 | 0.046448 |
| Protein Tyrosine Phosphatase, Receptor Type, U | PTPRU | 8.99 | 10.3 | -2.47 | 0.004646 |
| Ceramide Synthase 4 | CERS4 | 6.41 | 7.72 | -2.47 | 0.031966 |
| Zinc Finger Protein 292 | ZNF292 | 7.3 | 8.59 | -2.45 | 0.035548 |
| Transmembrane Protein 56 | TMEM56 | 6.21 | 7.5 | -2.45 | 0.047421 |
| Transforming, Acidic Coiled-Coil Containing Protein 1 | TACC1 | 9.66 | 10.95 | -2.45 | 0.022547 |
| Platelet Endothelial Aggregation Receptor 1 | PEAR1 | 7.12 | 8.4 | -2.44 | 0.042856 |
| Osteopetrosis Associated Transmembrane Protein 1 | OSTM1 | 6.68 | 7.97 | -2.44 | 0.037431 |
| Family With Sequence Similarity 102, Member A | FAM102A | 8.31 | 9.6 | -2.44 | 0.021348 |
| Meningioma (Disrupted In Balanced Translocation) 1 | MN1 | 6.83 | 8.11 | -2.43 | 0.005042 |
| ELOVL Fatty Acid Elongase 4 | ELOVL4 | 5.14 | 6.42 | -2.43 | 0.002186 |
| Delta-Like 1 (Drosophila) | DLL1 | 5.82 | 7.1 | -2.43 | 0.046843 |
| Ankyrin Repeat Domain 36B Pseudogene 1 | ANKRD36BP1 | 4.76 | 6.04 | -2.43 | 0.039098 |
| Tubulin, Alpha 1a | TUBA1A | 8.41 | 9.68 | -2.42 | 0.006271 |
| Dynein, Cytoplasmic 1, Heavy Chain 1 | DYNC1H1 | 8.59 | 9.86 | -2.41 | 0.020219 |
| Sorting Nexin 9 | SNX9 | 8.15 | 9.42 | -2.4 | 0.010356 |
| Autism Susceptibility Candidate 2 | AUTS2 | 5.93 | 7.19 | -2.4 | 0.009458 |
| Syntaxin Binding Protein 1 | STXBP1 | 9.31 | 10.57 | -2.39 | 0.00932 |
| DENN/MADD Domain Containing 5A | DENND5A | 8.35 | 9.61 | -2.39 | 0.019393 |
| Ubiquitin Protein Ligase E3 Component N-Recognin 4 | UBR4 | 8.73 | 9.98 | -2.36 | 0.00999 |
| Fibrinogen-Like 2 | FGL2 | 11.14 | 12.37 | -2.35 | 0.049138 |
| Protein Kinase Domain Containing, Cytoplasmic | PKDCC | 8.25 | 9.47 | -2.33 | 0.040776 |
| Prolyl Endopeptidase | PREP | 6.57 | 7.79 | -2.32 | 0.048802 |
| Cysteine-Rich Protein 2 | CRIP2 | 9.22 | 10.44 | -2.32 | 0.026883 |
| Sperm-Tail PG-Rich Repeat Containing 1 | STPG1 | 6.21 | 7.42 | -2.31 | 0.006554 |
| Tumor Necrosis Factor (Ligand) Superfamily, Member 10 | TNFSF10 | 8.71 | 9.91 | -2.3 | 0.02984 |
| RNA Binding Motif, Single Stranded Interacting Protein 3 | RBMS3 | 7.77 | 8.97 | -2.3 | 0.04194 |
| Exostosin Glycosyltransferase 1 | EXT1 | 9.8 | 11 | -2.29 | 0.0129 |
| Transmembrane Protein 106B | TMEM106B | 8.75 | 9.94 | -2.28 | 0.020497 |
| Nudix (Nucleoside Diphosphate Linked Moiety X)-Type Motif 11 | NUDT11 | 4.41 | 5.6 | -2.28 | 0.018775 |
| Mitochondrial Pyruvate Carrier 1 | MPC1 | 7.76 | 8.94 | -2.27 | 0.025186 |
| Mediator Complex Subunit 23 | MED23 | 7.94 | 9.13 | -2.27 | 0.022705 |
| Spermatogenesis Associated 6 | SPATA6 | 7.21 | 8.39 | -2.26 | 0.030583 |
| Sorting Nexin 14 | SNX14 | 7 | 8.18 | -2.26 | 0.010065 |
| SMAD Family Member 9 | SMAD9 | 8.91 | 10.09 | -2.26 | 0.011761 |
| Neurofibromin 1 Pseudogene 3; Neurofibromin 1 Pseudogene 5 | NF1P3; NF1P5 | 6.65 | 7.83 | -2.26 | 0.003717 |
| Neurofibromin 1 Pseudogene 3; Neurofibromin 1 Pseudogene 5 | NF1P3; NF1P5 | 6.65 | 7.83 | -2.26 | 0.003717 |
| Cytoplasmic Polyadenylation Element Binding Protein 2 | CPEB2 | 6.48 | 7.65 | -2.26 | 0.008426 |
| Kelch-Like Family Member 28 | KLHL28 | 7.01 | 8.18 | -2.25 | 0.047245 |
| Protein Tyrosine Phosphatase Type IVA, Member 2 | PTP4A2 | 10.08 | 11.24 | -2.24 | 0.024296 |
| Heme Binding Protein 2 | HEBP2 | 9.49 | 10.65 | -2.24 | 0.035928 |
| Potassium Voltage-Gated Channel, Shal-Related Subfamily, Member 3 | KCND3 | 5.14 | 6.29 | -2.22 | 0.012198 |
| SMAD Family Member 4 | SMAD4 | 9.02 | 10.16 | -2.21 | 0.027218 |
| Glutamine And Serine Rich 1 | QSER1 | 8.26 | 9.4 | -2.21 | 0.049826 |
| Signal-Induced Proliferation-Associated 1 Like 1 | SIPA1L1 | 10.38 | 11.52 | -2.2 | 0.006261 |
| IQ Motif Containing K | IQCK | 6.75 | 7.88 | -2.2 | 0.0058 |
| G Protein-Coupled Receptor 180 | GPR180 | 6.66 | 7.79 | -2.19 | 0.007596 |
| F-Box And Leucine-Rich Repeat Protein 4 | FBXL4 | 6.63 | 7.76 | -2.19 | 0.029441 |
| Dual Specificity Phosphatase 18 | DUSP18 | 5.74 | 6.87 | -2.19 | 0.040151 |
| Sortilin-Related VPS10 Domain Containing Receptor 2 | SORCS2 | 6.89 | 8.01 | -2.18 | 0.02355 |
| Phosphoprotein Enriched In Astrocytes 15 | PEA15 | 10.06 | 11.18 | -2.18 | 0.014887 |
| Phosphatidylinositol-4-Phosphate 3-Kinase, Catalytic Subunit Type 2 Alpha | PIK3C2A | 9.51 | 10.63 | -2.18 | 0.049407 |
| Origin Recognition Complex, Subunit 3 | ORC3 | 7.44 | 8.57 | -2.18 | 0.048548 |
| Heterogeneous Nuclear Ribonucleoprotein U-Like 2; HNRNPUL2-BSCL2 Readthrough | HNRNPUL2; HNRNPUL2-BSCL2; RP11-831H9.16 | 8.08 | 9.21 | -2.18 | 0.026287 |
| Dnaj (Hsp40) Homolog, Subfamily C, Member 6 | DNAJC6 | 4.92 | 6.04 | -2.18 | 0.010284 |
| Prickle Homolog 2 (Drosophila) | PRICKLE2 | 6.57 | 7.69 | -2.17 | 0.012086 |
| Nucleoporin 43kda | NUP43 | 8.39 | 9.51 | -2.17 | 0.039843 |
| Argonaute RISC Catalytic Component 3 | AGO3; EIF2C3 | 7.18 | 8.29 | -2.17 | 0.043506 |
| Argonaute RISC Catalytic Component 2 | AGO2; EIF2C2 | 7.38 | 8.5 | -2.17 | 0.025978 |
| Transmembrane Protein 25 | TMEM25 | 7.05 | 8.16 | -2.16 | 0.003834 |
| TEK Tyrosine Kinase, Endothelial | TEK | 6.66 | 7.77 | -2.16 | 0.017054 |
| Mex-3 Homolog C (C. Elegans) | MEX3C | 7.83 | 8.94 | -2.16 | 0.005976 |
| Matrix-Remodelling Associated 5 | MXRA5 | 6.65 | 7.75 | -2.15 | 0.002646 |
| Kiaa1009 | KIAA1009 | 4.62 | 5.72 | -2.15 | 0.005389 |
| Kiaa1462 | KIAA1462 | 7.87 | 8.97 | -2.14 | 0.009671 |
| Ubiquitin-Conjugating Enzyme E2, J1 | UBE2J1 | 8.54 | 9.63 | -2.13 | 0.0229 |
| Matrix-Remodelling Associated 8 | MXRA8 | 10.4 | 11.49 | -2.13 | 0.000239 |
| Potassium Voltage-Gated Channel, Shab-Related Subfamily, Member 1 | KCNB1 | 6.39 | 7.47 | -2.12 | 0.042265 |
| Zinc Finger, SWIM-Type Containing 5 | ZSWIM5 | 6.76 | 7.83 | -2.11 | 0.000262 |
| PAP Associated Domain Containing 5 | PAPD5 | 6.98 | 8.05 | -2.11 | 0.018589 |
| Apolipoprotein L, 1 | APOL1 | 6.35 | 7.43 | -2.11 | 0.031448 |
| SNF2 Histone Linker PHD RING Helicase, E3 Ubiquitin Protein Ligase | SHPRH | 6.55 | 7.62 | -2.1 | 0.045467 |
| Transmembrane Protein 50A | TMEM50A | 10.6 | 11.67 | -2.09 | 0.015122 |
| SERTA Domain Containing 4 | SERTAD4 | 5.45 | 6.52 | -2.09 | 0.036747 |
| Round Spermatid Basic Protein 1 | RSBN1 | 7.16 | 8.22 | -2.09 | 0.023791 |
| Kiaa1377 | KIAA1377 | 6.29 | 7.35 | -2.08 | 0.02285 |
| Formin 2 | FMN2 | 4.53 | 5.59 | -2.08 | 0.007923 |
| Diacylglycerol Kinase, Epsilon 64kda | DGKE | 5.07 | 6.13 | -2.08 | 0.009783 |
| 5-3 Exoribonuclease 1 | XRN1 | 8.28 | 9.33 | -2.08 | 0.038232 |
| Growth Differentiation Factor 10 | GDF10 | 6.32 | 7.37 | -2.07 | 0.01379 |
| Lectin, Galactoside-Binding, Soluble, 1 | LGALS1 | 10 | 11.04 | -2.06 | 0.031043 |
| Thioredoxin-Like 4A | TXNL4A | 8.49 | 9.52 | -2.05 | 0.041725 |
| FAT Atypical Cadherin 1 | FAT1 | 7.79 | 8.82 | -2.05 | 0.010786 |
| Tenascin XA (Pseudogene) | TNXA | 8.5 | 9.52 | -2.03 | 0.04049 |
| Synapse Differentiation Inducing 1 | SYNDIG1 | 8.83 | 9.85 | -2.03 | 0.030393 |
| Angiomotin | AMOT | 6.07 | 7.1 | -2.03 | 0.01316 |
| Low Density Lipoprotein Receptor-Related Protein 1 | LRP1 | 10.67 | 11.68 | -2.02 | 0.000122 |
| Discs, Large Homolog 3 (Drosophila) | DLG3 | 5.41 | 6.43 | -2.02 | 0.011919 |
| Blood Vessel Epicardial Substance | BVES | 7.67 | 8.69 | -2.02 | 0.04263 |
| Kinesin Family Member 1B | KIF1B | 7.24 | 8.25 | -2.01 | 0.00669 |
| Cysteine And Histidine-Rich Domain (CHORD) Containing 1 | CHORDC1 | 6.15 | 7.15 | -2.01 | 0.038901 |
