# Supplementary material for: Pleomorphism and drug resistant cancer stem cells are characteristic of aggressive primary meningioma cell lines
Source: Cancer Cell Int. 2017 Jul 21;17:72. doi: 10.1186/s12935-017-0441-7 (PMC5521079; doi:10.1186/s12935-017-0441-7)
Supplement: Supplementary file 4 — Additional file 4: Table S2. Predicted differential pathways generated by Ingenuity Pathway Analysis Software. The transcriptome comparison was carried out for group 1 (Tumors Jed49_MN, Jed36_MN) versus group 2 (Tumors Jed04_MN, Jed18_MN, Jed34_MN, Jed40_MN). [file 12935_2017_441_MOESM4_ESM.docx]

**Additional Table 2.** Predicted differential pathways generated by Ingenuity Pathway Analysis Software (IPA) between Group 1 (Tumours Jed49_MN, Jed36_MN) versus group 2 (Tumours Jed04_MN, Jed18_MN, Jed34_MN, Jed40_MN).

| **Top Canonical Pathways** | **p-value , Overlap** |
| --- | --- |
| MSP-RON Signaling Pathway | 3.38E-03, 8.7 % 4/46 |
| Role of NANOG in Mammalian Embryonic Stem Cell Pluripotency | 3.96E-03, 5.4 % 6/111 |
| Factors Promoting Cardiogenesis in Vertebrates | 8.15E-03, 5.4 % 5/92 |
| Human Embryonic Stem Cell Pluripotency | 9.73E-03, 4.5 % 6/134 |
| GABA Receptor Signaling | 1.28E-02, 6.0 % 4/67 |
| **Top Upstream Regulator** | **p-value of overlap** |
| ESR1 | 9.29E-06 |
| Rosiglitazone | 1.02E-05 |
| HTT | 5.49E-05 |
| Dexamethasone | 5.79E-05 |
| CTNNB1 | 1.15E-04 |
| **Diseases and Disorders** | **p-value , #Molecules** |
| Cancer | 1.35E-02 - 6.56E-12, 253 |
| Gastrointestinal Disease | 1.31E-02 - 6.56E-12, 207 |
| Organismal Injury and Abnormalities | 1.35E-02 - 6.56E-12, 254 |
| Reproductive System Disease | 1.35E-02 - 2.04E-08, 122 |
| Neurological Disease | 1.35E-02 - 1.76E-06, 83 |
| **Molecular and Cellular Functions** | **p-value , #Molecules** |
| Cellular Movement | 1.25E-02 - 1.03E-07, 63 |
| Cell Death and Survival | 1.28E-02 - 8.48E-05, 96 |
| Cell Cycle | 1.23E-02 - 8.54E-05, 14 |
| Cellular Development | 1.34E-02 - 1.02E-04, 84 |
| Cellular Growth and Proliferation | 1.34E-02 - 1.02E-04, 44 |
| **Physiological System Development and Function** | **p-value , #Molecules** |
| Embryonic Development | 1.35E-02 - 1.02E-04, 50 |
| Organismal Development | 1.35E-02 - 1.02E-04, 79 |
| Tissue Development | 1.34E-02 - 1.62E-04, 68 |
| Nervous System Development and Function | 1.35E-02 - 1.81E-04, 51 |
| Cardiovascular System Development and Function | 1.35E-02 - 2.59E-04, 50 |
| **Assays: Clinical Chemistry and Hematology** | **p-value , #Molecules** |
| Levels of Alkaline Phosphatase | 2.55E-03 - 2.55E-03 5 |
| Increased Levels of ALT | 2.27E-01 - 2.27E-01 1 |
| **Cardiotoxicity** | **p-value , #Molecules** |
| Cardiac Dysfunction | 3.35E-01 - 3.98E-03, 5 |
| Cardiac Necrosis/Cell Death | 3.16E-01 - 9.01E-03, 9 |
| Cardiac Proliferation | 1.02E-01 - 1.09E-02, 5 |
| Cardiac Arteriopathy | 3.99E-02 - 1.10E-02, 10 |
| Cardiac Arrythmia | 5.45E-01 - 1.35E-02, 7 |
| **Hepatotoxicity** | **p-value , #Molecules** |
| Liver Hyperplasia/Hyperproliferation | 5.58E-01 - 2.26E-04, 111 |
| Hepatocellular Carcinoma | 2.98E-01 - 9.74E-03, 17 |
| Liver Necrosis/Cell Death | 3.44E-01 - 2.66E-02, 7 |
| Biliary Hyperplasia | 2.68E-02 - 2.68E-02, 1 |
| Liver Fibrosis | 4.72E-01 - 3.50E-02, 5 |
| **Nephrotoxicity** | **p-value , #Molecules** |
| Renal Necrosis/Cell Death | 4.11E-01 - 3.23E-02 12 |
| Glomerular Injury | 2.93E-01 - 3.99E-02 4 |
| Renal Fibrosis | 2.93E-01 - 3.99E-02 2 |
| Kidney Failure | 2.17E-01 - 7.82E-02 5 |
| Renal Inflammation | 1.00E00 - 7.82E-02 5 |
| **Top Networks** |  |
| ID Associated Network Functions | **Score** |
| Cancer, Cell Death and Survival, Organismal Injury and Abnormalities | 42 |
| Skeletal and Muscular System Development and Function, Tissue Morphology, Lipid Metabolism | 40 |
| Cellular Movement, Cardiovascular System Development and Function, Cell-To-Cell Signaling and Interaction | 40 |
| Developmental Disorder, Hereditary Disorder, Neurological Disease | 29 |
| Cancer, Organismal Injury and Abnormalities, Cellular Development | 27 |
| **Top Tox Lists** | **p-value , Overlap** |
| Hepatic Fibrosis | 2.24E-03, 6.1 % 6/99 |
| Increases Damage of Mitochondria | 9.20E-03, 18.2 % 2/11 |
| Cardiac Necrosis/Cell Death | 1.06E-02, 3.4 % 9/267 |
| Cardiac Fibrosis | 1.37E-02, 3.7 % 7/187 |
| Increases Transmembrane Potential of Mitochondria and Mitochondrial Membrane | 2.98E-02 6.0 % 3/50 |
| **Fold Change up-regulated top molecules, Exp. Value** | **Fold Change down-regulated top molecules, Exp. Value** |
| RELN, 27.130 | CYP4B1 -17.400 |
| CALB1, 23.540 | FBXO32 -15.980 |
| KLRC4, 13.670 | MEDAG -10.940 |
| AGR2, 12.660 | SERPINA3 -8.950 |
| SLC22A8, 10.970 | MFAP5 -8.380 |
| KLRC4-KLRK1/KLRK1*, 9.190 | UCHL1 -8.250 |
| NRG1, 7.850 | LRRN1 -8.250 |
| SLC16A12, 7.240 | PRRX1 -8.040 |
| SNRPN* , 5.910 | LPHN2 -8.020 |
| IGF2, 5.330 | KCNMA1 -8.010 |
