# Supplementary material for: Pleomorphism and drug resistant cancer stem cells are characteristic of aggressive primary meningioma cell lines
Source: Cancer Cell Int. 2017 Jul 21;17:72. doi: 10.1186/s12935-017-0441-7 (PMC5521079; doi:10.1186/s12935-017-0441-7)
Supplement: Supplementary file 5 — Additional file 5: Figure S3. CSCs markers expression in situ. A) Images for immunofluorescence co-staining of stem cell markers CD133+Sox2+ (Green, Red) or Nestin+Ki67+ (Green, Red) and AGR2+ BMI1+ (Red, Green) in low grade (Jed62_MN) and high grade (Jed45_MN) tumors. B) Mean percentages of co-positive cells. Error bars represent count errors between three independent regions within each tissue. All images were taken at 20×. [file 12935_2017_441_MOESM5_ESM.pptx]

## Slide 1
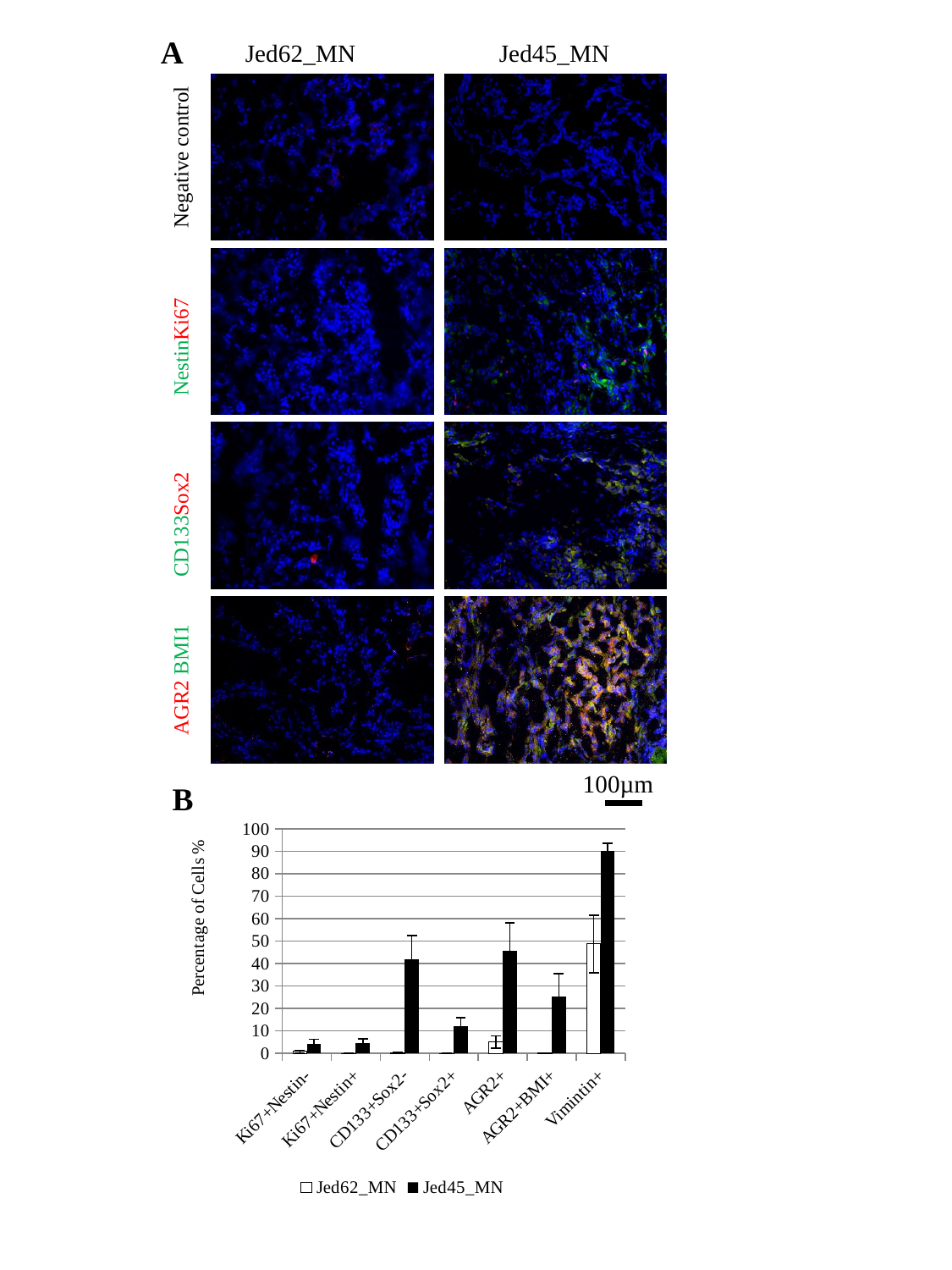

A
Jed62_MN
Jed45_MN
Negative control
NestinKi67
CD133Sox2
AGR2 BMI1
100µm
B
### Chart
| Category | Jed62_MN | Jed45_MN |
|---|---|---|
| Ki67+Nestin- | 0.7824644099227068 | 4.311957799745816 |
| Ki67+Nestin+ | 0.0 | 4.442812772690516 |
| CD133+Sox2- | 0.2363319255661099 | 41.80897049546302 |
| CD133+Sox2+ | 0.0 | 12.025197809792827 |
| AGR2+ | 5.015547350177253 | 45.566006857751994 |
| AGR2+BMI+ | 0.14836795252225546 | 25.134096731910915 |
| Vimintin+ | 48.72921866150157 | 90.17906912511418 |
