# Supplementary material for: Pleomorphism and drug resistant cancer stem cells are characteristic of aggressive primary meningioma cell lines
Source: Cancer Cell Int. 2017 Jul 21;17:72. doi: 10.1186/s12935-017-0441-7 (PMC5521079; doi:10.1186/s12935-017-0441-7)
Supplement: Supplementary file 6 — Additional file 6: Figure S4. AGR2 co-expression with CSCs markers in situ. A) Images for immunofluorescence co-staining of stem cell markers Nestin+AGR2+(Green, Red), CD133+AGR2+(Green, Red), and Sox2+AGR2+ (Green, Red) in low grade (Jed62_MN and Jed40_MN) and high grade (Jed49_MN and Jed45_MN) tumors. B) Mean percentages of co-positive cells. Error bars represent count errors between three independent regions within each tissue. All images were taken at 20×. [file 12935_2017_441_MOESM6_ESM.pptx]

## Slide 1
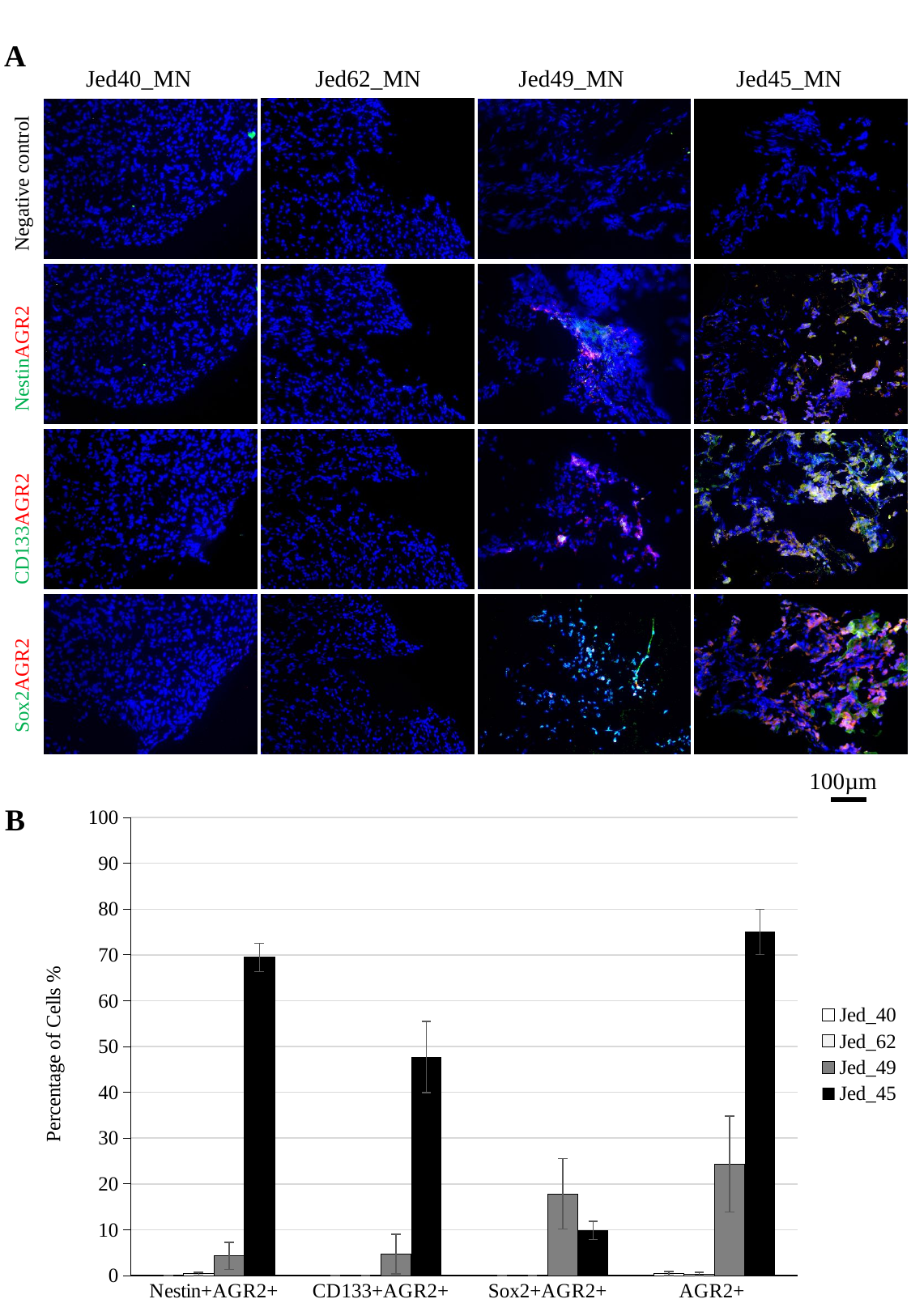

A
Jed40_MN
Jed62_MN
Jed49_MN
Jed45_MN
Negative control
NestinAGR2
CD133AGR2
Sox2AGR2
100µm
B
### Chart
| Category | Jed_40 | Jed_62 | Jed_49 | Jed_45 |
|---|---|---|---|---|
| Nestin+AGR2+ | 0.0 | 0.3844809507165327 | 4.306912775968151 | 69.46135831381733 |
| CD133+AGR2+ | 0.0 | 0.07010164738871363 | 4.67706013363029 | 47.72628843655262 |
| Sox2+AGR2+ | 0.0 | 0.0 | 17.84679089026915 | 9.883449883449885 |
| AGR2+ | 0.4464285714285714 | 0.3433208489388264 | 24.380600867615243 | 75.02935178561808 |
