# Supplementary material for: Pleomorphism and drug resistant cancer stem cells are characteristic of aggressive primary meningioma cell lines
Source: Cancer Cell Int. 2017 Jul 21;17:72. doi: 10.1186/s12935-017-0441-7 (PMC5521079; doi:10.1186/s12935-017-0441-7)
Supplement: Supplementary file 7 — Additional file 7: Figure S5. Average percentages of morphologies counts for Jed62_MN and Jed79_MN. Each tumor was divided into four portions that were grown in either DMEM-F12 +10% FBS (Blue line), or DMEM high glucose concentrations of 4500 mg/L (Gibco) +10% FBS (Grey line), or DMEM high glucose concentrations of 4500 mg/L (Gibco) + 5% FBS (Yellow line), or DMEM low glucose concentrations of 1000 mg/L (Gibco) + 10% FBS (Orange line). Horizontal accesses represent morphologies described in Figure 2a (M: M Type, N: N Type, O:G: O Type, G: G Type, A: A Type, and D: D Type). A minimum of 500 cells were counted per week per cell line, per condition. [file 12935_2017_441_MOESM7_ESM.pptx]

## Slide 1
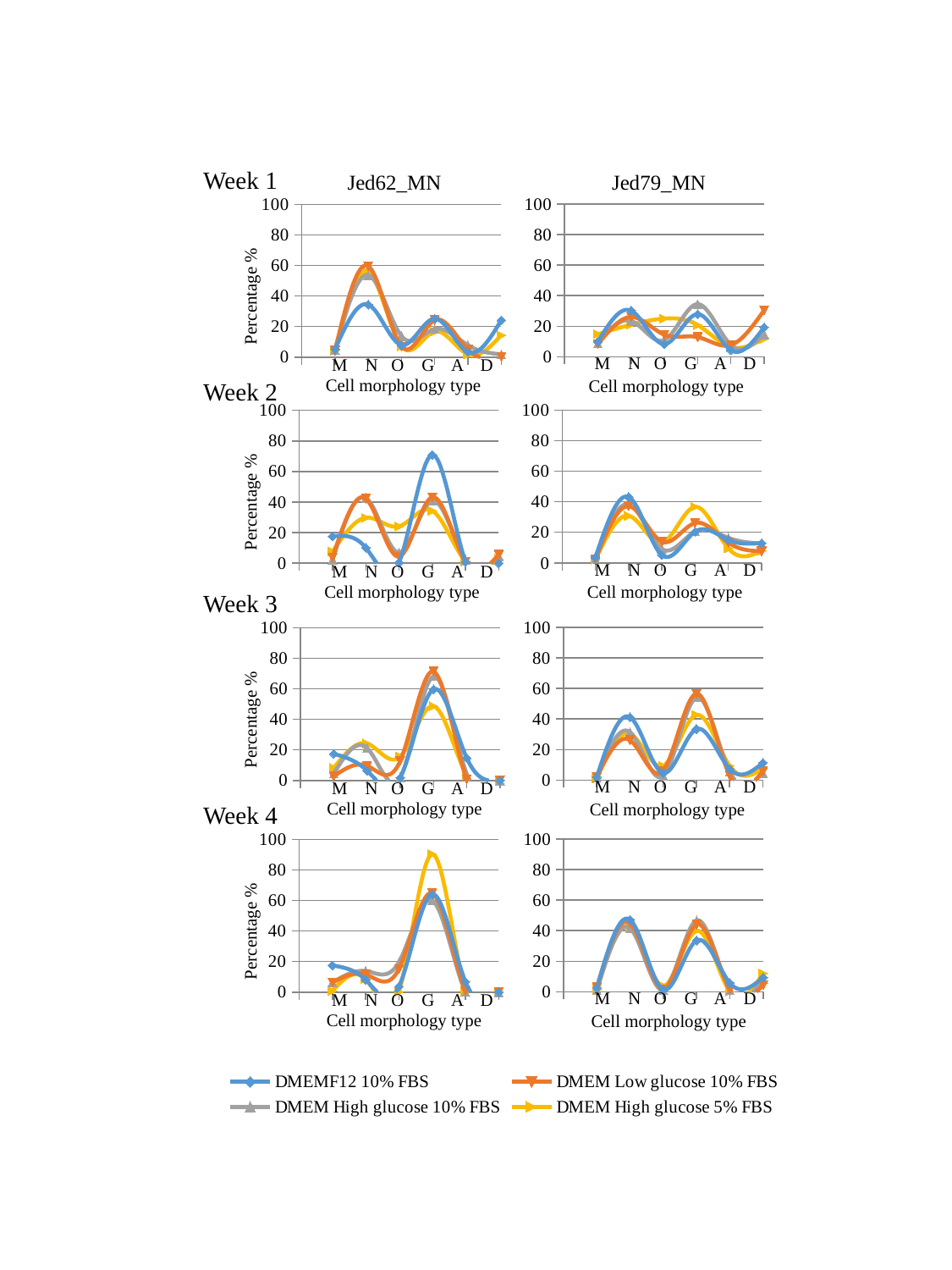

Week 1
Jed62_MN
Jed79_MN
### Chart
| Category | DMEMF12 10% FBS | DMEM Low glucose 10% FBS | DMEM High glucose 10% FBS | DMEM High glucose 5% FBS |
|---|---|---|---|---| M N O G A D
Cell morphology type
### Chart
| Category | DMEMF12 10% FBS | DMEM Low glucose 10% FBS | DMEM High glucose 10% FBS | DMEM High glucose 5% FBS |
|---|---|---|---|---| M N O G A D
Cell morphology type
Percentage %
Week 2
### Chart
| Category | DMEMF12 10% FBS | DMEM Low glucose 10% FBS | DMEM High glucose 10% FBS | DMEM High glucose 5% FBS |
|---|---|---|---|---| M N O G A D
Cell morphology type
### Chart
| Category | DMEMF12 10% FBS | DMEM Low glucose 10% FBS | DMEM High glucose 10% FBS | DMEM High glucose 5% FBS |
|---|---|---|---|---| M N O G A D
Cell morphology type
Percentage %
Week 3
### Chart
| Category | DMEMF12 10% FBS | DMEM Low glucose 10% FBS | DMEM High glucose 10% FBS | DMEM High glucose 5% FBS |
|---|---|---|---|---| M N O G A D
Cell morphology type
### Chart
| Category | DMEMF12 10% FBS | DMEM Low glucose 10% FBS | DMEM High glucose 10% FBS | DMEM High glucose 5% FBS |
|---|---|---|---|---| M N O G A D
Cell morphology type
Percentage %
Week 4
### Chart
| Category | DMEMF12 10% FBS | DMEM Low glucose 10% FBS | DMEM High glucose 10% FBS | DMEM High glucose 5% FBS |
|---|---|---|---|---| M N O G A D
Cell morphology type
### Chart
| Category | DMEMF12 10% FBS | DMEM Low glucose 10% FBS | DMEM High glucose 10% FBS | DMEM High glucose 5% FBS |
|---|---|---|---|---| M N O G A D
Cell morphology type
Percentage %
### Chart:
| Category | DMEMF12 10% FBS | DMEM Low glucose 10% FBS | DMEM High glucose 10% FBS | DMEM High glucose 5% FBS |
|---|---|---|---|---|
